# Entanglement-Stabilized Nanoporous Polymer Films Made by Mechanical Deformation

**DOI:** 10.1021/acs.macromol.4c00187

**Published:** 2024-03-14

**Authors:** Hsiao-Ping Hsu, Kurt Kremer

**Affiliations:** Max-Planck-Institut für Polymerforschung, Ackermannweg 10, Mainz 55128, Germany

## Abstract

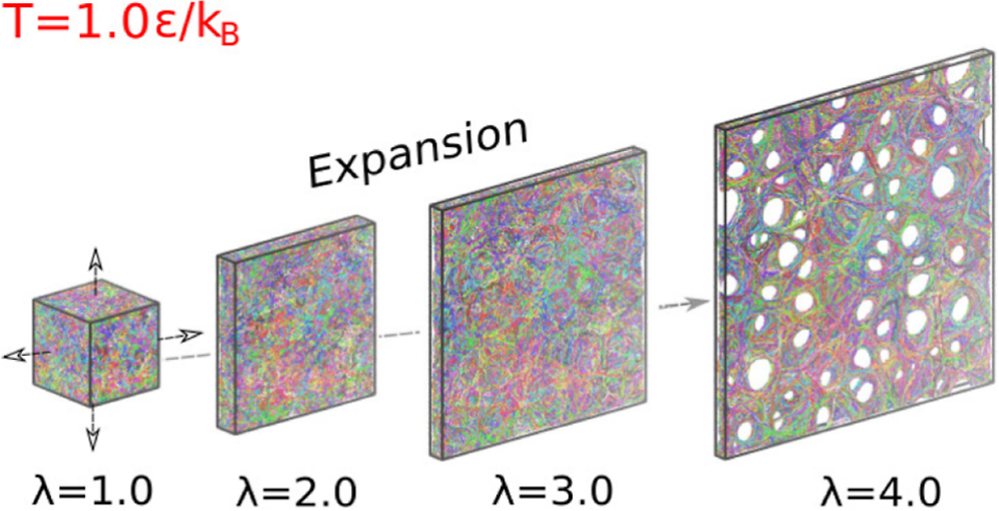

We present a new simulation-guided process to create nanoporous materials, which does not require specific chemical treatment and solely relies on mechanical deformation of pure highly entangled homopolymer films. Starting from fully equilibrated freestanding thick polymer melt films, we apply a simple “biaxial expansion” deformation. Upon expansion holes form, which are prevented from growing and coalescing beyond a characteristic size due to the entanglement structure of the melt. We investigate the local morphology, the void formation upon expansion, and their stabilization. The dependence of the average void (pore) size and void fraction (porosity) on the total strain and subsequent relaxation is investigated. Furthermore, the stabilization of the porous structure of the thin expanded films through cooling below the glass transition temperature *T*_g_ is discussed.

## Introduction

Thin porous polymer films have attracted much attention due to their many potential applications ranging from supporting media in tissue engineering to separation processes. For example, they can be used as a temporary biodegradable framework for organ and tissue regeneration, advanced water treatment, gas separation, medical, or food technology. Because of such versatile applications, porous materials have become an important research topic for decades. A brief introduction of different synthesis methods of generating micro-, meso-, and macro-porous materials with different properties is given in ref (1). There exist also several reviews describing specific application-driven requirements such as specific large absorption areas, high surface to volume ratios,^2−4^ and prospective developments.^5^ Typically, the preparation of (nano)porous films takes advantage of incompatibility of components. Not surprisingly, block copolymers^6^ have been widely employed for designing nanostructured porous materials by varying molecular weight, block volume fractions, thermodynamic incompatibility of the blocks, and solvent qualities.^3,4,7−10^ Examples are bottle brush polymer blocks,^11^ silk-like proteins with triblock architectures,^12,13^ or a metal–organic framework (MOF) compounds.^14,15^ All these processes of synthesizing porous materials are based on phase separation of different blocks/components, where at least one component is chemically removed to create pores. Other commonly used methods of producing porous polymer membranes are via fast evaporation of solvent of deformed polymer solution films,^16^ e.g., GoreTex, and evaporation-induced phase separation^17^ to prepare porous silicon rubber membranes with tunable pore size for biomedical applications. However, all above-mentioned processes of making porous membranes are rather complex, often needing very elaborate precision chemistry and requiring processes controlled in detail. We here take a different approach and propose a simple strategy to make thin porous films based on dry, highly entangled polymer melts without any additives.

The idea is to view entanglements of very long chains in a polymer melt not as a complication, which, e.g., causes processing difficulties but as a feature to be employed to make specific structured materials with new properties. In an earlier work, we have shown that highly entangled polymer melts subject to elongation under certain conditions can lead to materials, which are homogeneous at a first glance but contain areas of clustered entanglement points.^18,19^ Such a transient structure can easily be stabilized by cooling the systems below the glass transition temperature *T*_g_. Thus, one arrives at a chemically homogeneous melt with spatially inhomogeneous viscoelastic properties. Here, we take a different route to employ entanglements in a polymer melt to create a new material. Failure of polymer materials under strain is considered to be connected to void formation and their subsequent growth, see, e.g., ref (20). This growth is facilitated by chain motion and the presence of chain ends. Chain ends originate either from finite chain length or are created by chain breaking. Chain end motion results in a release of topological constraints and subsequent growth of voids. The goal is to perform a strong mechanical deformation, i.e., here a biaxial expansion, which does not allow for unbounded growth of voids. That should result in a porous material, where the size of the pores remains nanoscopically small. For that, we start with a thick, equilibrated film of a highly entangled polymer melt. For estimates and simulations described below,^21^ we use melts of chain length *N* = 2000 ≈ 72*N*_e_, *N*_e_ ≈ 28 monomers being the entanglement chain length,^22,23^ and monodisperse polystyrene PS melts with *M* = 10^6^ Da ≈ 60*M*_e_, *M*_e_ ≈ 16,600 Da being the entanglement molecular weight.^24^ This film is expanded biaxially while the third dimension is free to adjust. The expansion rate is fast compared to the inverse terminal relaxation rate of the chains and even faster compared to the Rouse time of the chains but slow compared to relaxation processes of the order of the entanglement length, i.e., on the scale of the reptation tube diameter (see the Supporting Information). By that, we expect the chains to globally follow the deformation of the sample affinely, while locally relaxation prevents the built up of high strain, which could cause chain scission. Topological constraints originating from the noncrossability of the chains, to a first approximation, are also conserved. In turn, this means that in course of the expansion, any small void that forms upon the induced strain only can grow as much as is permitted by the approximate conservation of entanglement constraints. Our proposal is that this should lead to controlled nanoporous materials, which could be stabilized by quenching them well below *T*_g_. In refs (25 and 26), we have demonstrated that a stable monodisperse nanoporous PS film can be easily created following a protocol analogous to molecular dynamics (MD) simulations to produce nanoporous films. Based on the concept of reptation tube diameters, we show a semiquantitative agreement of scattering function between simulation and experiment. While this was a very short account of the proposed procedure, we here give a detailed account on the underlying simulations to create nanoporous films.

The outline of this first detailed paper is as follows: we first shortly review the main features of the semiflexible bead–spring model and the preparation of thick, freestanding films of highly entangled polymer melts and the proposed deformation protocol in the next section. In the third section, we investigate the morphological properties of different expanded films. Then, the development of porous structures of thin expanded films upon subsequent relaxation in the fourth section and cooling in the fifth section are analyzed. Section six finally contains our conclusions. The subsequent work will discuss in detail the physical properties of the stabilized nanoporous films and also compare them to experiments on films produced from entangled polystyrene melts.

## Simulation Setup

### Simulation Model and Equilibration

**Figure 1:**
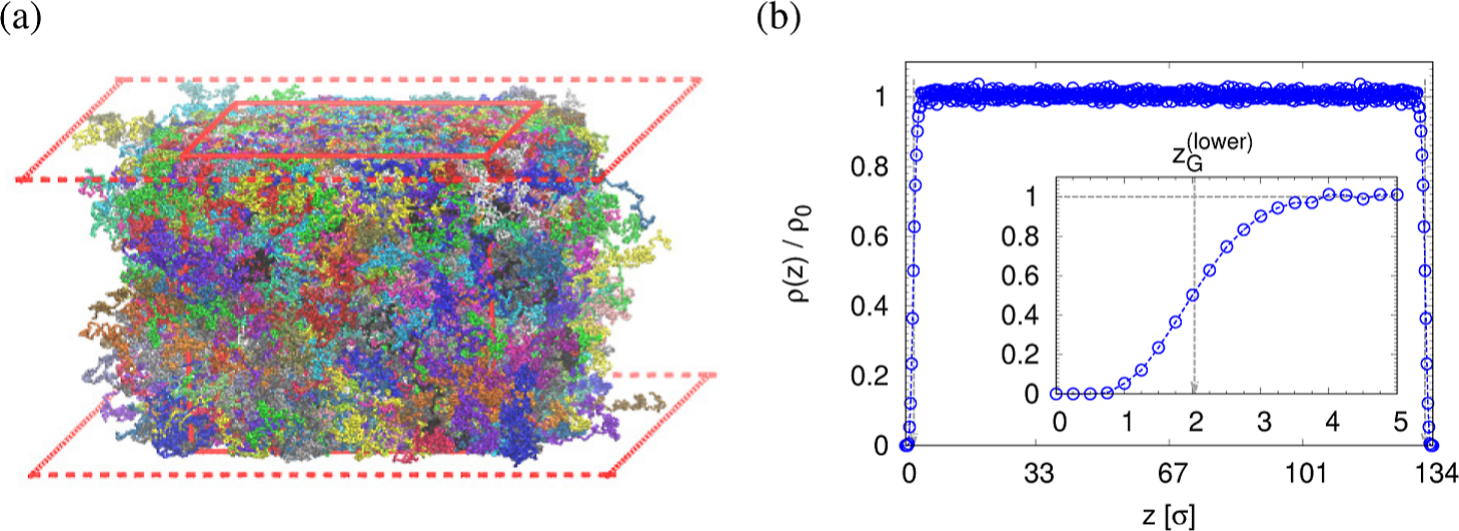
(a) Snapshot of an equilibrated (unperturbed) freestanding thick film of *h* ≈ 130σ, containing *n*_c_ = 1000 chains of *N* = 2000 monomers, at temperature *T* = 1.0ϵ/*k*_B_ and pressure *P* = 0.0ϵ/σ^3^ right after confining walls are switched off and relaxed for . Periodic boundary conditions in the *x* and *y* directions are applied. (b) Monomer density profile ρ(*z*) in the direction perpendicular to the interfaces of film shown in (a). Data for *z* < 5σ close to the surface are shown in the inset. The lower interface of the film located at *z*_G_^(lower)^ is indicated by an arrow.

We start with fully equilibrated thick freestanding films of highly entangled polymer melts. Based on two variants of the bead–spring model,^27−32^ we have developed an efficient way to equilibrate large polymer melt films^21^ by a hierarchical backmapping methodology similar to that for bulk polymer melts.^33^ For this approach, equilibration times scale linearly with system size, independent of chain length. We here study films containing *n*_c_ = 1000 weakly semiflexible polymer chains of *N* = 2000 monomers at the bulk melt (monomer number) density ρ_0_ = 0.85σ^–3^. Model parameters are chosen that the entanglement length^22,23^*N*_e_ = 28. Thus, our films contain a highly entangled melt. A snapshot of a starting system, a freestanding film of film thickness *h* ≈ 130σ ≈ 4.3*R*_g_^(0)^ (*R*_g_^(0)^ ≈ 30.15σ being the root mean square radius of gyration of the chains in bulk) is shown in Figure 1a. The two lateral dimensions are *L*_w_ = *L*_*x*_ = *L*_*y*_ ≈ 134σ. As shown in Figure 1b, by the monomer density profile ρ(*z*) in the direction perpendicular to the interfaces of film, melt density is assumed all over the interior of the film up to a very thin interface layer (around 1σ) with the environment. Based on ρ(*z*), the effective film thickness *h* = *z*_G_^(upper)^ – *z*_G_^(lower)^, *z*_G_^(upper)^ = 131.95σ and *z*_G_^(lower)^ = 2.03σ being the two planar interfaces of films, is determined using the concept of Gibbs dividing surfaces.^21,34−36^

To prepare freestanding films by the hierarchical backmapping procedure, at each level, a melt of chains between two confining, short-range repulsive walls at a distance of *L*_*z*_ with periodic boundary conditions parallel to the walls (*x*, *y* directions) is equilibrated at density ρ = *n*_c_*N*/(*L*_*x*_*L*_*y*_*L*_*z*_) ≈ ρ_0_ = 0.85σ^–3^. The interaction with the structureless repulsive walls is given by a 10–4 LJ planar wall potential^37,38^*U*_wall_(*z*) with a wall monomer interaction strength at ε_w_ = 0.0032ϵ. By that, the monomer density ρ(*z*) ≈ ρ_0_ is kept even next to the walls.^21,39^ (Note that the resulting thickness of the freestanding film is rather insensitive to the precise value of .) This initial equilibration is performed for the standard semiflexible bead spring model, which only contains repulsive interactions between nonbonded beads, which results in a pressure *P* ≈ 5ϵ/σ^3^. Furthermore, the usually applied bending interaction^29,40,41^ is not well suited to study low temperatures because of unphysical chain stretching at low temperatures. At that point, we switch to a recently developed variant,^31,32^ see the Supporting Information. There, a short-ranged attractive interaction *U*_ATT_(*r*) is introduced so that the pressure of a bulk polymer melt *P* ≈ 0.0ϵ/σ^3^ at density ρ = 0.85σ^–3^ and temperature *T* = 1.0ϵ/*k*_B_. The chain stiffness is controlled by the new bond-bending potential *U*_BEND_(θ), so that as in experiment, chain conformations are only very weakly temperature-dependent. At *T* = 1.0ϵ/*k*_B_, conformations are indistinguishable from the ones with the conventional bending potential with *k*_θ_ = 1.5ϵ, allowing a seamless switch of models. With this new variant of the model, we first run the confined film in the constant number, volume, temperature (*NVT*) ensemble for about  where τ_e_ = τ_0_*N*_e_^2^ ≈ 2266τ with τ_0_ ≈ 2.89τ is the entanglement time^23^ followed by the constant number, pressure, temperature (*NPT*) ensemble for another  to ensure that all three diagonal terms of the pressure tensor *P*_*xx*_ = *P*_*yy*_ = *P*_*zz*_ ≈ 0.0ϵ/σ^3^ before the walls are removed. For this model, the glass transition temperature *T*_g_ in bulk and for thick films is about^42^ 0.67ϵ/*k*_B_. Details of calculating the pressure tensor *P*_αβ_ with α, β = *x*, *y*, *z* is given in the Supporting Information.

The ESPResSo++ package^43,44^ is used to perform the standard MD simulations of polymer films with a Langevin thermostat in the *NVT* ensemble, while in the *NPT* ensemble, a Hoover barostat is used together with a Langevin thermostat.^45,46^ For production runs, the MD time step is set to Δ*t* = 0.01τ and the friction coefficient Γ = 0.5τ^–1^. All simulations are performed at temperature *T* = 1.0ϵ/*k*_B_ before the deformed films are stabilized at lower temperatures. The list of symbols used in the paper is given in Table 1.

**Table 1:** List of Symbols Used in the Paper

Lennard-Jones units			strain rate	strain
energy [ϵ]	length [σ]	time [τ]	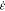	λ

### Deformation Process: Biaxial Expansion

**Figure 2:**
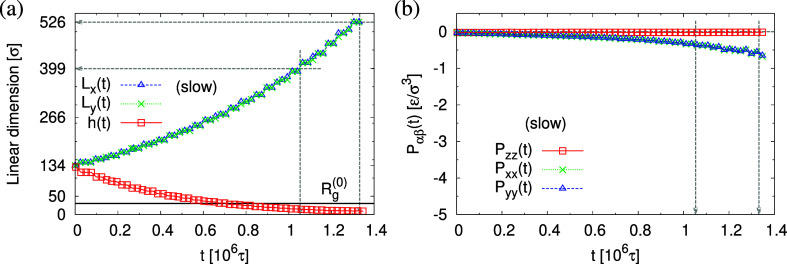
Time series of two lateral dimensions *L*_*x*_(*t*), *L*_*y*_(*t*), and thickness *h*(*t*) of film (a), and three diagonal terms of pressure tensor *P*_α,β_(*t*) (b) for films subject to biaxial expansion at *T* = 1.0ϵ/*k*_B_ upon deformation with . Values of dimensions and pressure for expansion ratios of λ = *L*_*x*,*y*_/*L*_w_ ≈ 3.0 and 4.0, *L*_*x*_(*t*) = *L*_*y*_(*t*) ≈ 399σ and 526σ, respectively, are indicated by arrows.

We apply a simple “biaxial expansion” deformation to polymer films as shown in Figure 1a. The films are stretched into two lateral dimensions, i.e., equi-biaxial strain^47−49^ with periodic boundary conditions up to a maximum expansion of 4 × 4 at *T* = 1.0ϵ/*k*_B_, where the thickness of the film is free to adjust, cf. Figure 2a. The components of pressure tensor along the two lateral dimensions are the same during the expansion process as shown in Figure 2b. Technically, the film is first instantaneously stretched by a factor of 1.02 along the *x*-direction and then along the *y*-direction. This deformation step is chosen to be small enough so as not to induce any numerical instabilities. After each deformation step along one direction, the lateral dimensions are fixed and the system is allowed to relax for (0.02τ_R_/*C*), resulting in an effective strain rate . *C* is the measure of the strain rate relative to the Rouse time τ_R_ of the chains. We initially use *C* = 77, i.e., the strain rate is much faster compared to the Rouse relaxation of the overall chains, while subchains of chain length up to about  are expected to be able to relax during deformation. Moreover, after each expansion step, the film thickness relaxes and the instant pressure, *P*_*zz*_, quickly approaches zero as shown in Figure 2. This latter relaxation required even a slowing down of the expansion, leading to an effective *C* ≈ 24 based on the total deformation and the total time for deformation and thus leading to , see the Supporting Information for the detailed simulation protocol. This protocol is repeated until the desired expansion is reached. In our simulation, expanded polymer films finally are in the thin-film regime (*h* < *R*_g_^(0)^) with *P*_*zz*_ ≈ 0.0ϵ/σ^3^.

As mentioned before, we aim at conserving the density of entanglement constraints during deformation, at least approximately. In the reptation model, chains require the Rouse time τ_R_ ∼ *N*^2^ for equilibration within the tube followed by the diffusive motion out of the tube, which needs the disentanglement time of τ_d_ ∼ *N*^3.4^. The deformation has to be fast enough to avoid the latter. Having an experimental realization in mind, this is easily achievable by adjusting the temperature distance from the glass transition temperature *T*_g_. On the more microscopic side, the deformation should be slow enough to allow for local monomer packing equilibration in order to avoid a situation, which in experiment could lead to chain scission. For that, the relevant time scale is the entanglement length Rouse time τ_e_ = τ_R_(*N*_e_). At *T* = 1.0ϵ/*k*_B_, for the present model, τ_e_ ≈ 2266τ, while τ_R_ ≈ 11.6 × 10^6^τ. This led to our final choice of *C* = 24 for slow deformation. Time series of pressure and linear dimensions of freestanding films upon expansion are shown in Figure 2.

We also tested a very fast deformation initially starting at *C* = 32,000. However, there some short waiting periods had to be introduced to allow *P*_*zz*,*xx*,*yy*_ to adjust (see the Supporting Information), leading to effectively *C* = 14,710 (fast), allowing for a relaxation of subchains of only ≈0.6*N*_e_ ≈ 17 monomers. Applied to PS, even such a fast expansion would allow for local relaxations of chains of *M*_w_ of about 9960 Da or 96 repeat units and should not affect the local amorphous packing of atactic PS significantly.^24^ Data for fast expansion are shown in Figure S4 of the Supporting Information. As *L*_*x*_(*t*) and *L*_*y*_(*t*) increase, the elastic restoring force of the film results in negative pressure values for *P*_*xx*_(*t*), *P*_*yy*_(*t*). Since the film thickness is allowed to adjust freely, *P*_*zz*_(*t*) remains at about 0ϵ/σ^3^ and shows only for fast deformation a small negative dip (see the Supporting Information). With increasing strain, the faster strain rate leads to a larger negative pressure (retraction force), as expected. Eventually, in the thin-film regime (*h* < *R*_g_^(0)^), the effective film thickness *h*(*t*) approximately reaches a plateau value and *P*_*zz*_(*t*) remains at 0.0ϵ/σ^3^.

### Morphological Properties in Response to Biaxial Deformation

**Figure 3:**
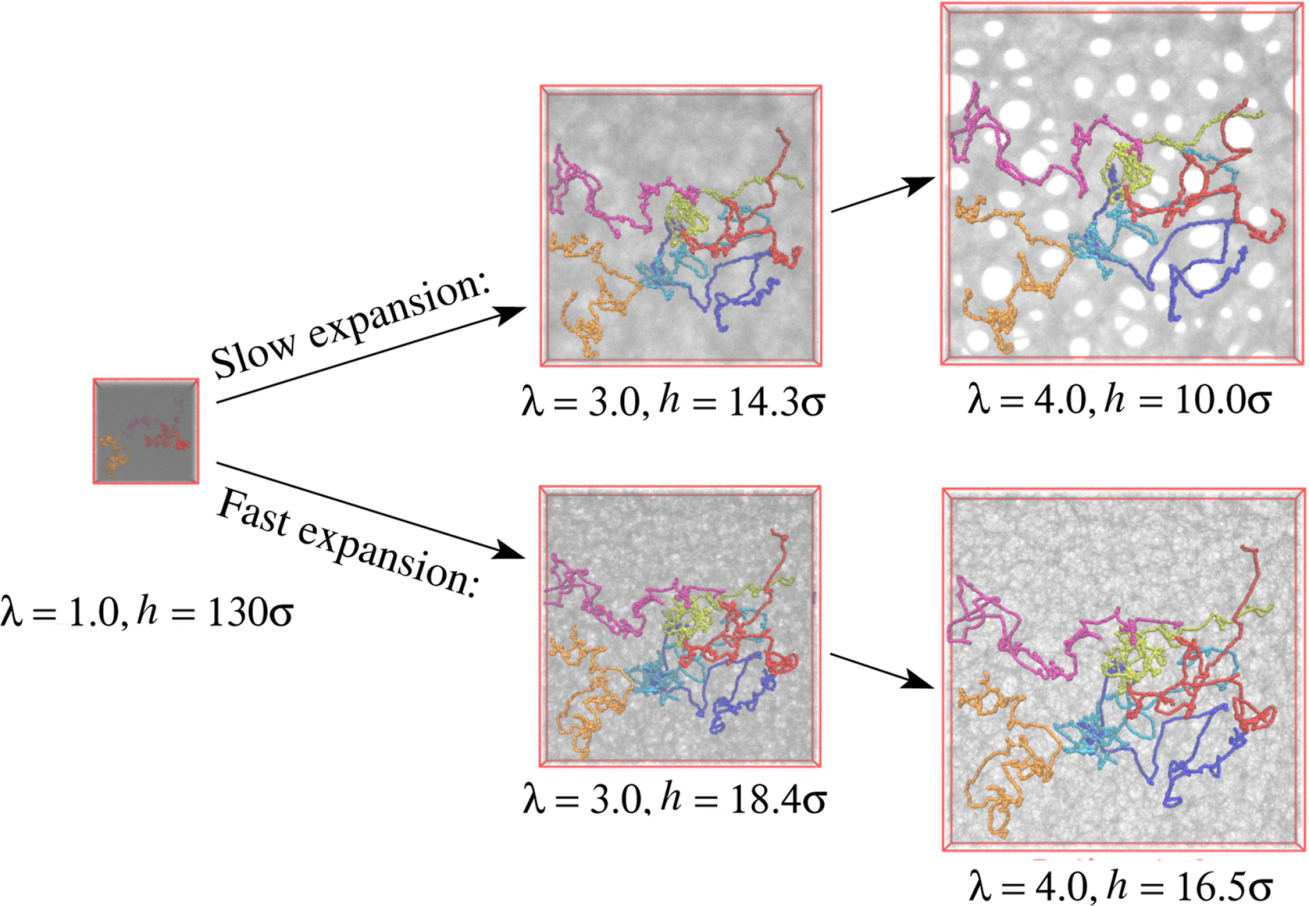
Snapshots of freestanding films subject to slow and fast expansion at strain of λ = 1.0, 3.0, and 4.0 as indicated. The corresponding film thicknesses *h* are also given for comparison. The same six randomly selected chains are marked for a better illustration of conformational changes.

Snapshots of freestanding films at λ = *L*_*x*,*y*_/*L*_w_ ≈ 1.0, 3.0, and 4.0 right after slow and fast expansion without further relaxation are shown in Figure 3. To follow conformational changes, six randomly selected chains in the films are shown as well. Obviously, morphologies of expanded freestanding films depend on strain rate  and strain (stretching ratio) λ. For slow stretching, we observe initial void (pore) formation, while for fast stretching, only strong density variations are visible. Already at a first glance, there is a striking similarity of the conformations of the marked chains for fast and slow expansion, and the in-plane deformation of the chains appears to roughly follow the box deformation. Note that the films laterally cannot shrink due to in-plane periodic boundary conditions. It is clearly visible that the chains extend over several pores with a spherical-like cross section, which prevents their coalescence. Details of internal structures of expanded films at λ ≈ 3.0 and 4.0 are shown in Figures S6–S9 for comparison. Illustration of the process of a typical void formation is shown in Figure S10.

### Individual Chain Conformations

Due to the biaxial expansion, the linear dimensions of chain conformations characterized by the mean square end-to-end distance  and the mean square radius of gyrations  are decomposed into two components parallel and perpendicular to the stretching directions,  where  and . Here, ⟨··· ⟩_λ_ stands for the average over all *n*_c_ = 1000 chains for films at strain λ.

**Figure 4:**
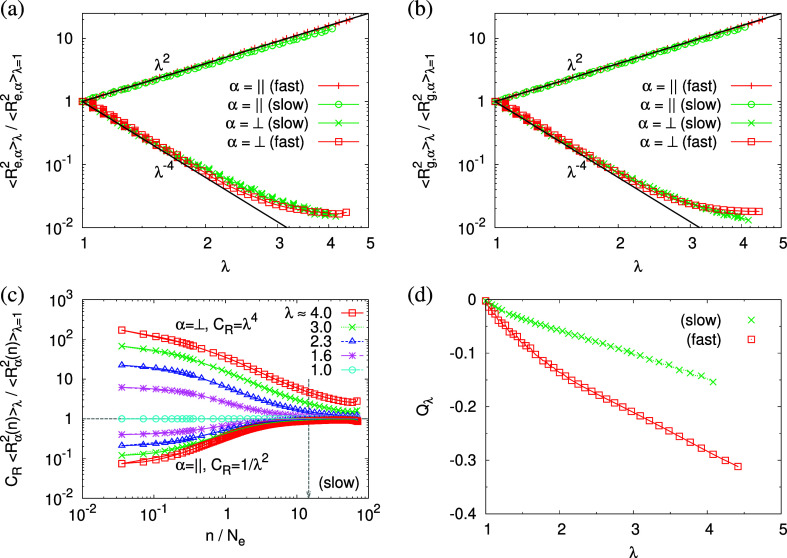
(a,b) Two components of rescaled mean square end-to-end distance,  (a) and rescaled mean square radius of gyration,  (b), plotted versus the strain of λ. (c) Two components of rescaled mean square internal distance, , plotted versus the rescaled chemical distance *n*/*N*_e_. (d) Bond orientation order parameter *Q*_λ_ plotted versus the strain of λ. Data are for films expanded at two different strain rates, as indicated. In (a–c), α = ∥ and ⊥ denote the components in the direction parallel and perpendicular to the expanding directions, respectively. Reference values are  and  in (a) and  and  in (b). In (a,b), affine scaling laws are shown by straight lines for comparison. In (c), five strain values of λ are chosen, as indicated, and subchains of chain length *n* = 14.6*N*_e_ estimated from the corresponding strain rate  are pointed out by an arrow.

If the chain conformations would deform affinely with the film expansion, entanglement constraints would automatically be conserved, as anticipated by our concept. For this, we first examine the strain-dependent chain expansion normalized by the unperturbed freestanding film values,  and , respectively, as shown in Figure 4. In the expansion plane , indicating that chains globally follow the film expansion affinely. In the perpendicular direction, chains deform affinely (λ^–4^) only up to about λ ≈ 1.6, leveling off at weakly strain-rate-dependent values. This only is possible if the overall averaged density (including, e.g., voids) in the film is reduced, as already indicated by Figure 3. We also include the results of the rescaled internal mean-square distances right after deformation (*C* = 24) at several selected strains of λ,  in Figure 4c, normalized by the affine deformation scaling parameter *C*_R_, as indicated. Here, *n* is the chemical distance between two bonds along the path of the same chain. As originally proposed, for the in-plane expansion, chain conformations follow the affine deformation on lengths scales above about *n* = 10*N*_e_, which roughly agrees with the estimate based on *C* = 24. Even further down to about 5 to 6 *N*_e_, the deviation from the proposed affine scaling is very small. For the perpendicular components, deviations are in agreement with reduced overall density, as already indicated by the end-to-end distances. For fast expansion (see Figure S5), subchains of chain length above 4*N*_e_ follow affine deformation, which is significantly longer than the predicted length scale 0.6*N*_e_ for *C* = 14,710. This shows that initial chains conformation response follows rather different initial pathways due to topological constraints compared to the slowly expanded films. These global conformational changes also lead to characteristic changes in the bond orientational order parameter *Q*_λ_. Choosing the *z*-axis as a reference,  where ϕ_*z*_ is the angle between any bond vector and the *z*-axis. For an isotropic distribution of bond directions *Q*_λ_ = 0, while *Q*_λ_ = −1/2, if all bonds would lie in the *xy* plane. Figure 4d shows that with increasing strain, bond vectors approach the in-plane orientation. This effect is more pronounced upon fast expansion.

**Figure 5:**
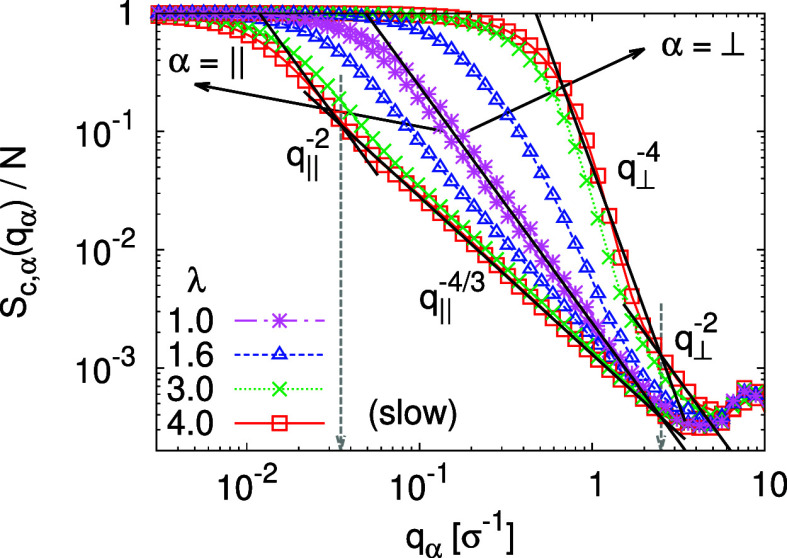
Two components of single chain structure factor *S*_c,α_(*q*) in the directions parallel (α = ∥) and perpendicular (α = ⊥) to the expansion plane for selected values of λ and *C* = 24. Theoretically predicted scaling laws are shown by straight lines for comparison, cf. text.

The conformational anisotropy of the chains also is directly visible in the single chain structure factor *S*_c_(*q*). As before, we here also distinguish  and *S*_c,⊥_(*q*_⊥_ = *q*_*z*_). The strain-dependent two components *S*_c,∥_(*q*_∥_) and *S*_c,⊥_(*q*_⊥_) are shown in Figure 5. Initially, chains in the unperturbed film behave as ideal chains, *S*_c,∥_(*q*_∥_) ∼ *q*_∥_^–2^ and *S*_c,⊥_(*q*_⊥_) ∼ *q*_⊥_^–2^, an indication that we are really dealing with thick films. As λ increases, *S*_c,∥_(*q*_∥_) decreases while *S*_c,⊥_(*q*_⊥_) increases, according to the Guinier law and consistent with the changes of  and  shown in Figure 4, respectively. As *S*_c,∥_(*q*_∥_) indicates beginning with about λ ≥ 3.0, chains are already highly stretched along the expanding directions. On shorter and intermediate length scales, *S*_c,∥_(*q*_∥_) ∼ *q*_∥_^–4/3^ is observed, indicating the appearance of 2-dimensional excluded volume like correlations between beads and voids. On large length scales (*q*_∥_ < 0.035σ^–1^), *S*_c,∥_(*q*_∥_) ∼ *q*_∥_^–2^ remains, indicating an expanded Gaussian chain, in agreement with the affine deformation proposal, cf. Figure 4. Perpendicular to the expansion plane, on short length scales (*q*_⊥_ > 2.5σ^–1^), chains still behave as ideal chains, *S*_c,⊥_(*q*_⊥_) ∼ *q*_⊥_^–2^. On larger scales, approaching the film thickness, a Porod law-like scaling *S*_c,⊥_(*q*_⊥_) ∼ *q*_⊥_^–4^ is observed for λ ≥ 3, indicating a sharp interface. For much thicker films of *h* ≈ 51σ at λ ≈ 1.6, the latter is not observed. Similar scaling behavior is also observed for the film subject to fast expansion.

### Porosity and Pore Size Distribution

**Figure 6:**
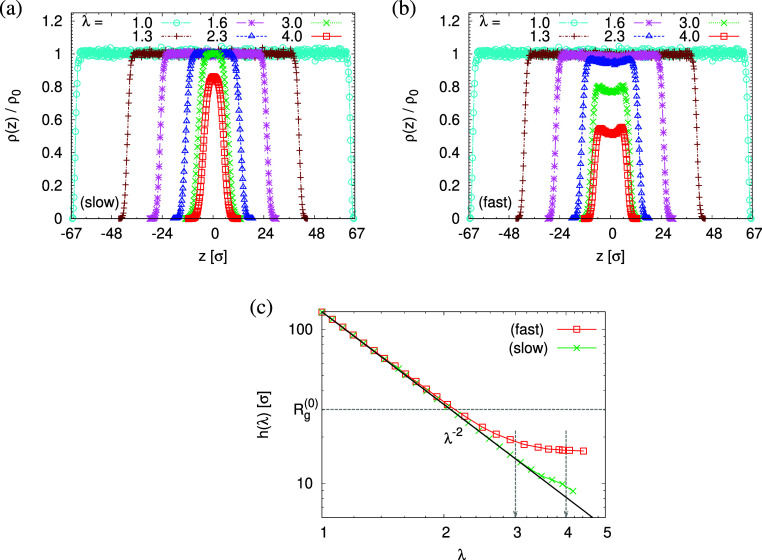
(a,b) Monomer density profiles rescaled by the bulk melt density, ρ(*z*)/ρ_0_, plotted as a function of *z* along the direction perpendicular to the interfaces of films subject to slow (a) and fast (b) expansion at several selected strain values of λ. (c) Film thickness *h*(λ) plotted versus strain of λ for films subject to slow and fast expansion, as indicated. In (a,b), the centers of expanded freestanding films in the *z*-direction are matched at *z* = 0σ. The two selected strains of λ ≈ 3.0 and 4.0 are indicated by arrows in (c).

To estimate the porosity ϕ and pore size distribution *P*(*D*_pore_) of pore diameter *D*_pore_ in expanded films, we adopt the definition given by Gubbins et al.,^50−53^ where ϕ and *P*(*D*_pore_) depend on the accessible volume of a hard spherical test particle of size 1.0σ; i.e., the test particles explore all regions in the film, where the nearest monomer is at least a distance of 1σ away. This is a purely geometrical measure, as no interaction between test particles and monomers is considered. The porosity ϕ is then the percentage of void volume *V*_void_ compared to the total effective volume *V*_film_ = *hL*_*x*_*L*_*y*_ of the films. The effective film thickness *h* is determined from the monomer density distribution ρ(*z*) as mentioned in the last section, see Figures 1 and 6. Similarly, the individual pore size *D*_pore_ in the film is determined by inserting a test particle of diameter *D*_pore_. A detailed description of estimating ϕ, *D*_pore_, and maximum pore size *D*_pore_^(max)^ by Monte Carlo simulations is given in Section SIV of the Supporting Information.

**Figure 7:**
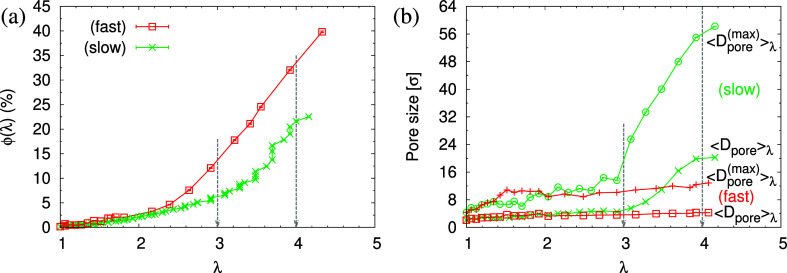
Porosity ϕ(λ) (a), mean maximum pore size , and mean pore size ⟨*D*_pore_⟩_λ_ (b), plotted versus the strain λ for films subject to slow and fast expansion, as indicated. Two selected strain of λ ≈ 3.0 and 4.0 are indicated by arrows.

The corresponding porosities ϕ(λ), average pore sizes , and maximum pore sizes , plotted versus strain of λ are shown in Figure 7. Obviously, the porosity ϕ(λ) in the expanded films increases monotonically with increasing strain λ. For small deformation (λ < 2.3), ϕ(λ) is independent of strain rate although small fluctuations are seen for the fast expanded film even though film thicknesses *h*(λ) follow the affine deformation, see Figure 6c. Beyond λ = 2.3, the porosity ϕ(λ) increases faster for fast expansion, in agreement with the weaker reduction of *h*(λ). This fits to the change in overall density ρ, which reduces stronger for fast deformation (Figure 6). At the same time, larger pores are formed in the slowly expanded film. Interestingly, we observe a tendency toward a plateau value of *h*(λ) beyond λ = 3, which depends on the strain rate . However, immediately after deformation, the film thickness *h*(λ) decays while no significant change in  is observed, see the next section.

**Figure 8:**
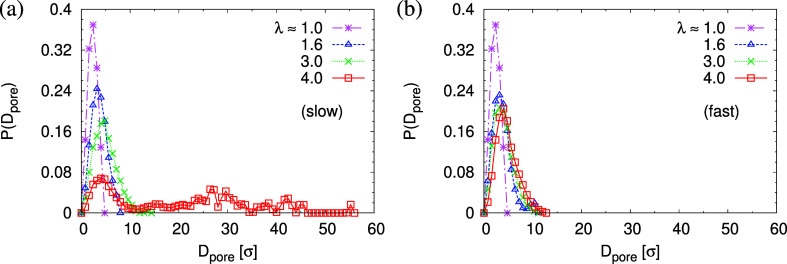
Pore size distributions *P*(*D*_pore_) plotted as a function of *D*_pore_ for films subject to slow (a) and fast (b) expansion at several selected strain values of λ. Curves for λ = 1.0 show the microscopic packing density fluctuation on scales of the order of the monomer size.

This option to adjust the pore size by the expansion process is also demonstrated by the pore size distribution *P*(*D*_pore_) as shown in Figure 8. Slow expansion to a strain of λ = 4 results in a broad distribution of pore sizes, *P*(*D*_pore_), while in contrast, only small but many more pores are detected for films subject to fast expansion, see Figures S8 and S9 for details. For small deformation, ρ(*z*) in the interior of expanded film remains at the bulk value ρ_0_, indicating that chain deformation still is too small to affect the packing, and no voids are formed. Our computer experiments suggest that the interplay of deformation rate and competing relaxation allows us to control the initial porosity ϕ and the pore sizes *D*_pore_ in films.

### Scattering Functions of Expanded Films

Scattering functions of inhomogeneous materials give additional insight, which can directly be compared to scattering experiments. For characterizing the structure of expanded films, we separately analyze the in-expansion-plane scattering functions, *S*_∥_(*q*_∥_), and scattering perpendicular to the film surfaces, *S*_⊥_(*q*_⊥_). Setting  with *n*_1_, *n*_2_ = 0, ±1, ±2, ... we get
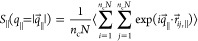
1where  is the vector between monomer *i* and monomer *j* in the film along the direction parallel to the expanding directions. In the same way
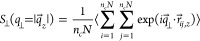
2

Note that for *S*_⊥_(*q*_⊥_), *q*_⊥_ does not assume discrete values, which would make *q* commensurable with the film thickness *h*. The normalization is set that *S*_⊥,∥_(*q*_⊥,∥_ = 0σ^–1^) = *n*_c_*N*.

**Figure 9:**
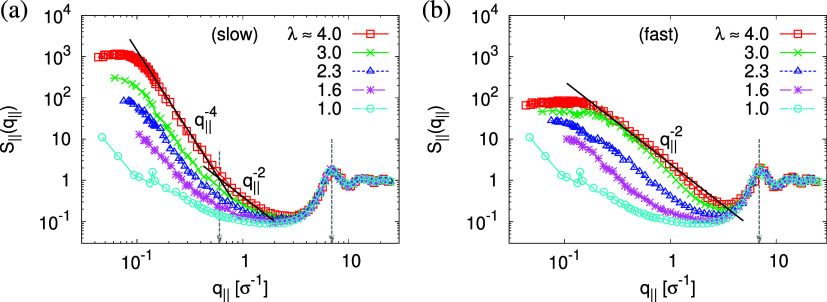
Collective structure factor *S*_∥_(*q*_∥_) in the direction parallel to the expanding directions, plotted versus the wave factor *q*_∥_ for films subject to slow (a) and fast (b) expansion. Theoretical predictions are shown by solid straight lines for comparison.

Upon expansion, *S*_∥_(*q*_∥_) increases with λ on large and intermediate length scales, while on short length scales, *q*_∥_ > 2σ^–1^, it remains unchanged, showing that the local monomer packing is conserved, cf. Figure 9. The peak at *q*_∥_^*^ ≈ 6.9σ^–1^, the so-called amorphous halo for amorphous materials, is a signature of the intermonomer packing distance of 2π/*q*_∥_^*^ ≈ 0.91σ and the same as in unperturbed bulk melts.^22,31^ From ideally sharp and smooth (flat) pore surfaces, one expects (Porod’s law) *S*_∥_(*q*) ∼ *q*_∥_^–4^ in the intermediate range of *q*_∥_.^54^ For slowly expanded films at 4.0, we observe a tendency toward such smooth void–polymer interfaces (see Figure S8, Supporting Information), which is qualitatively in agreement with experimental findings,^25^ and a crossover to *S*_∥_(*q*_∥_) ∼ *q*_∥_^–2^ with the increase of *q*_∥_, when the individual chain structure becomes relevant. The latter is only clearly seen for fast expanded films. For fast expanded films, clearly defined interfaces are not observed in *S*_∥_(*q*_∥_), in agreement with the visual inspection of Figures S9 and 7b.

**Figure 10:**
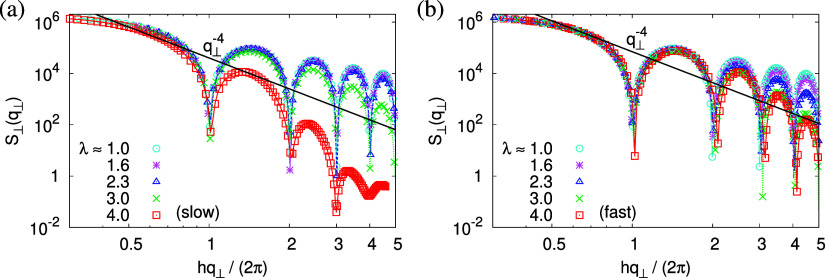
Collective structure factor *S*_⊥_(*q*_⊥_) in the direction perpendicular to the expanding direction, plotted versus the rescaled wave factor, *q*_⊥_/(2π/*h*(λ)), for films subjected to slow (a) and fast (b) expansion. The Porod law *S*_⊥_(*q*_⊥_) ∼ *q*_⊥_^–4^ is also shown by a straight line for comparison.

For *S*_⊥_(*q*_⊥_), the situation is more simple and *S*_⊥_(*q*_⊥_) can directly be used to measure the apparent film thickness *h* as shown in Figure 10. Rescaling the wave factor *q*_⊥_ by a factor of 2π/*h*(λ), we observe sharp minima at *n*_q_ = *hq*_⊥_/(2π), *n*_q_ = 1, 2, ... . With the increase of *q*_⊥_, the local minima slightly deviate from *n*_q_ for *n*_q_ > 1 due to small thickness variations of the films. However, the *q*^–4^ envelope of decay describing the sharp surface for expanded films at λ ≈ 3.0 and 4.0 in the thin film regime is not observed for both cases due to the void formation.

### Relaxation of Expanded Films after Deformation at *T* = 1.0ϵ/*k*_B_

**Figure 11:**
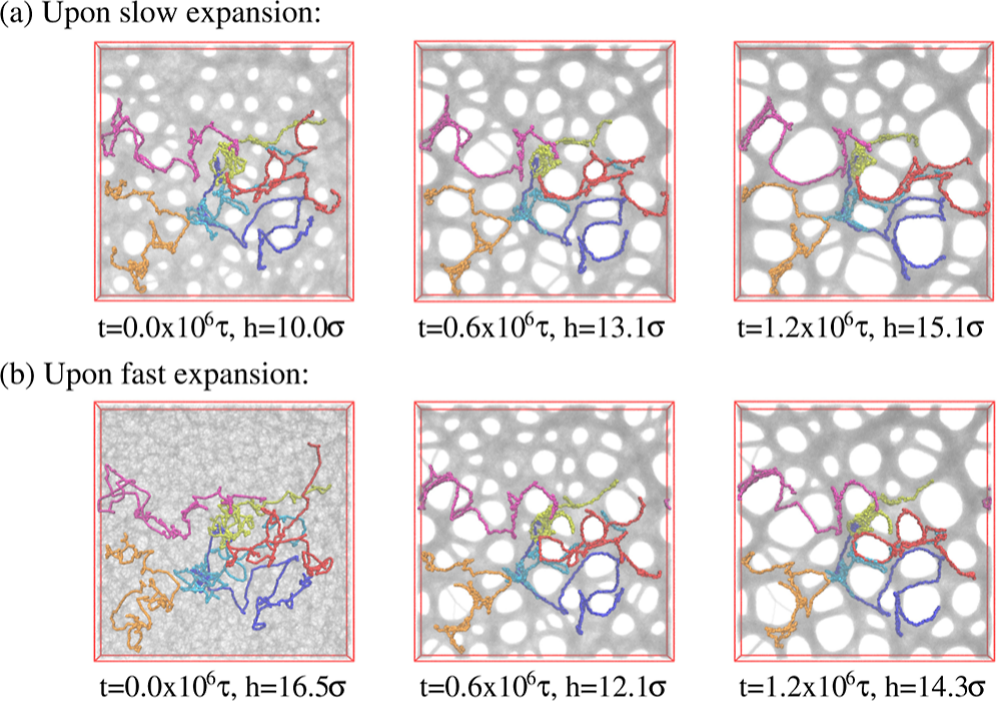
Snapshot configurations of thin porous films at λ ≈ 4.0 subject to relaxation for films upon slow (a) and fast (b) expansion at several selected relaxation times *t* and assumed thicknesses *h*, as indicated. The very same six chains as shown in Figure 3 are also marked for comparison.

So far, we have analyzed the initial properties of expanded highly entangled polymer films right after deformation at *T* = 1.0ϵ/*k*_B_, which is about 1.5*T*_g_^(0)^, *T*_g_^(0)^ ≈ 0.67ϵ/*k*_B_ being the glass transition temperature of a bulk polymer melt.^42,55^ The resulting instantaneous nonequilibrium structure of course is instable and the question arises whether relaxation at this high processing temperature would lead to immediate destabilization of the films. For that, we follow the time development of morphological changes of expanded thin films kept at strain λ ≈ 4.0. Figure 11 shows the changes for two samples, beginning right after slow and fast deformation, respectively.

Times covered range up to *t* = 1.2 × 10^6^ ≈ 530τ_e_ ≈ 0.1τ_R_, corresponding to the Rouse time of subchains of length of *N*_s_ ≈ 644. These snapshots lead to two striking observations. First, for the fast expanded film, initially, no well-defined pore structure is seen, which, after a very short relaxation time, starts to develop. The pore sizes increase accompanied by some drop in the number of pores. Furthermore, the pore structure appears to be quite similar to the one resulting from slow deformation. Since we start from the same initial melt film, the pore structure seems to be imprinted at least approximately in the melt morphology. The second observation is that the growth of the pores in both cases slows down significantly after about half of the relaxation time, which corresponds to the Rouse time of subchains of about 16*N*_e_. Pore sizes in the fast expanded sample appear only weakly smaller than in the slowly expanded system. This relaxation retardation is also demonstrated by the six marked chains whose conformations only marginally change, obviously due to topological constraints of highly entangled chains.^18,19,56^ More details of the increased bead friction will be published in a separate publication. Moreover, in all cases, the shape of pores tend to become spherical to minimize surface tension.

**Figure 12:**
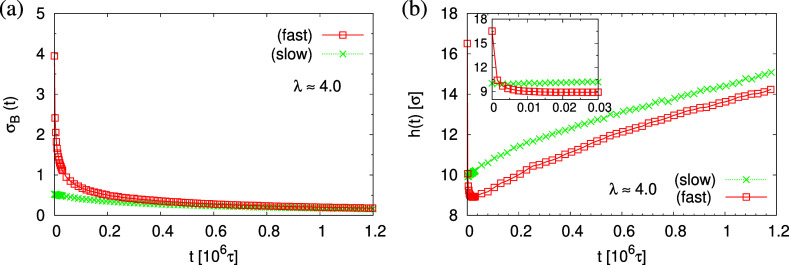
Net stress σ_B_(*t*) (a) and film thickness *h*(*t*) (b) plotted versus relaxation time *t* for porous films at λ ≈ 4.0 upon slow and fast expansion, as indicated. In the inset of (b), short time data for *t* < 0.03 × 10^6^τ are shown.

The finding that the system relaxation slows down significantly also is supported by the reduction of restoring forces per unit area, σ_B_(*t*), shown in Figure 12. Using eq S2, we observe a dramatic reduction in net restoring stress σ_B_(*t*) = |(*P*_*zz*_(*t*) – (*P*_*xx*_(*t*) + *P*_*yy*_(*t*))/2)| after an initial time of about (0.2–0.3) × 10^6^τ. At the same time, *P*_*zz*_(*t*) remains at 0.0ϵ/σ^3^. Furthermore, after that, the time-dependent stress is almost indistinguishable between the slow and fast expansion case, again in accord with the visual inspections of the membranes. The results of *h*(*t*) in Figure 12b show that for fast deformation, the initial reduction of thickness is more developed than for slow deformation and even crosses the thickness of the latter. Eventually, the fast expanded film even upon relaxation remains somewhat thinner in agreement with the apparently slightly smaller porosity and thus smaller pores.

**Figure 13:**
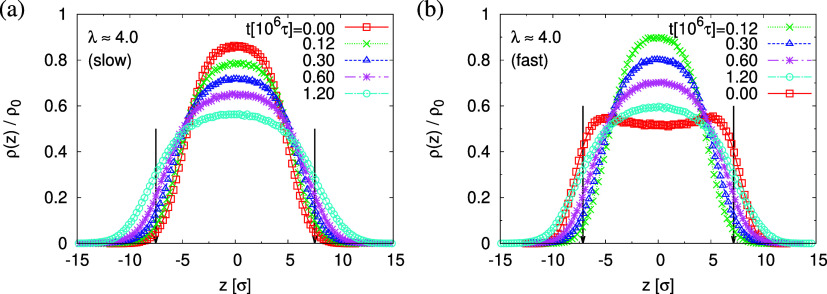
Scaled monomer density profiles, ρ(*z*)/ρ_0_, plotted as a function of *z* at λ ≈ 4.0 upon slow (a) and fast (b) expansion at several selected subsequent relaxation times *t*, as indicated. The centers of thin porous films in the *z*-direction are matched at *z* = 0σ. The interfaces located at *z*_G_^(lower)^ and *z*_G_^(upper)^ for films at *t* = 1.2 × 10^6^τ are indicated by arrows, respectively.

**Figure 14:**
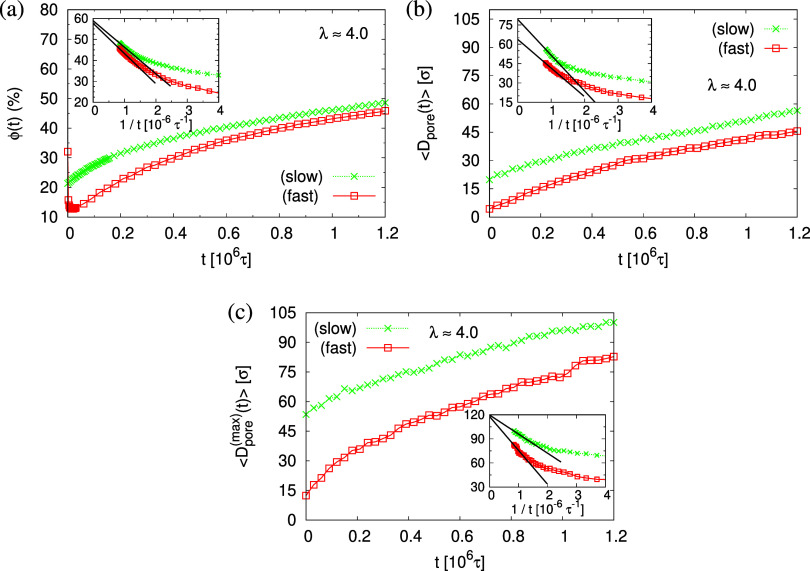
Porosity ϕ(*t*) (a), mean pore size ⟨*D*_pore_(*t*)⟩ (b), and mean maximum pore size ⟨*D*_pore_^(max)^(*t*)⟩ (c), plotted versus relaxation time *t* for porous films at λ ≈ 4.0 upon slow and fast expansion, as indicated. In the inset of (a–c), we plot the same data for *t* > 0.25 × 10^6^τ, respectively, versus 1/*t*. The straight lines indicate linear extrapolating of all data sets to *t* → ∞.

**Figure 15:**
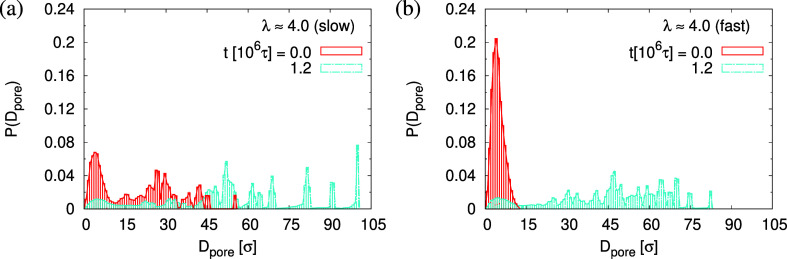
Histogram plot of pore size distribution *P*(*D*_pore_) for thin porous films at λ ≈ 4.0 upon slow (a) and fast (b) expansion. Only data for thin porous films at the subsequent relaxation times *t*/τ = 0 and 1.2 × 10^6^ are shown, as indicated.

This reverse behavior for the films upon fast expansion also appears in the change of monomer density profile ρ(*z*) (see Figure 13). The apparent difference in pore size and porosity is confirmed by the direct measurement of the porosity ϕ(*t*), the pore size *D*_pore_(*t*), and the maximum pore size *D*_pore_^(max)^(*t*), which all increase with time (see Figure 14). Again, for both cases, a (slight) slowing down of the relaxation with time *t* is observed. For both films, the average pore sizes and maximum pore sizes continue to grow only slowly in time. The average pore diameter at *t* = 1.2 × 10^6^τ corresponds to about 56σ ≈ 11*d*_T_ for slow expansion and 46σ ≈ 9*d*_T_ for the fast expansion case. Assuming a linear extrapolation to 1/*t* → 0, ϕ(*t*) ≈ 58% and ⟨*D*_pore_^(max)^(*t*)⟩ ≈ 120σ converge to the same values for both cases, while ⟨*D*_pore_(*t*)⟩ is larger for slow expansion, ⟨*D*_pore_(*t*)⟩ ≈ 80σ ≈ 16*d*_T_ (slow) and 64σ ≈ 13*d*_T_ (fast). The probability distributions of pore size *D*_pore_, *P*(*D*_pore_) are shown in Figure 15. At *t* = 0τ, *P*(*D*_pore_) has a unimodal distribution for the film upon fast expansion, and it becomes a much broader multimodal distribution at *t* = 1.2 × 10^6^τ. For the film upon slow expansion, results of *P*(*D*_pore_) show that the probability of finding larger pore size *D*_pore_ increases while it decreases for small pore size, as illustrated in Figure 11.

**Figure 16:**
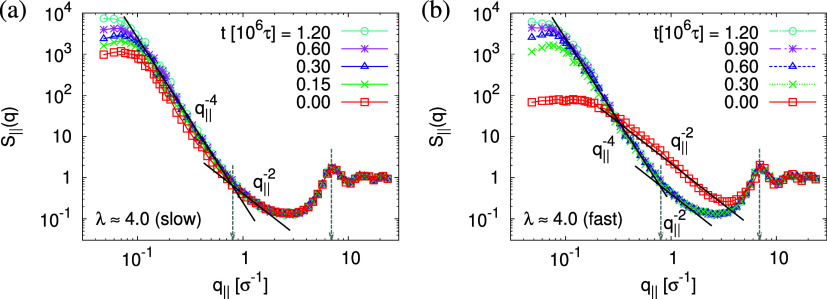
Collective structure factor *S*_∥_(*q*_∥_) in the directions parallel to the interface of thin porous films at λ ≈ 4.0 upon slow (a) and fast (b) expansion at several selected subsequent relaxation times *t*, as indicated. The theoretical predictions are also shown by solid lines for comparison. The positions of amorphous halo and crossover points are indicated by arrows.

The above-described scenario is well supported by the in expansion plane collective structure factor *S*_∥_(*q*_∥_) in Figure 16 at several relaxation times *t*. The region around the amorphous halo at *q*_∥_^*^ ≈ 6.9σ^–1^ remains unchanged upon relaxation for all times. Thus, the local bead packing is not affected by our processes. On larger scales, the signature of sharp pore surfaces, Porod scaling *S*_∥_(*q*_∥_) ∼ *q*_∥_^–4^, for slow deformation is stabilized and extended a little further to larger length scales with the increase of the porosity^57^ ϕ(*t*) (see Figure 14). For fast deformation, the initially fuzzy interfaces sharpen and already after short relaxation time, the same scaling is observed. The latter is essentially indistinguishable from the results for slow deformation. The initially large *q*^–2^ regime narrows down to a small region, just as for slow deformation. In all cases, *S*_∥_(*q*_∥_) reaches a shallow maximum/plateau at low *q*_∥_, roughly corresponding to distances around 100σ, corresponding to 2–3 average pore diameters and reminding us of a semidilute 2-d liquid of hard disks (i.e., the pores).

**Figure 17:**
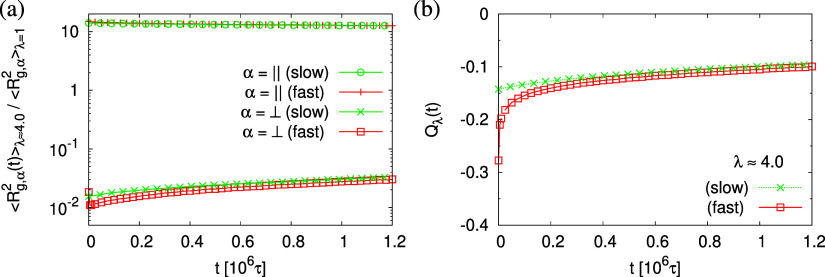
Two components of rescaled mean square radius of gyration ⟨*R*_g,α_^2^(*t*)⟩/⟨*R*_g,α_^2^(*t* = 0)⟩ (a) in the directions parallel (α = ∥) and perpendicular (α = ⊥) to the expending directions, and bond orientational order parameter *Q*_λ_(*t*) (b), plotted versus relaxation time *t*. Data are for thin porous films at λ ≈ 4.0 upon slow and fast expansion, as indicated.

**Figure 18:**
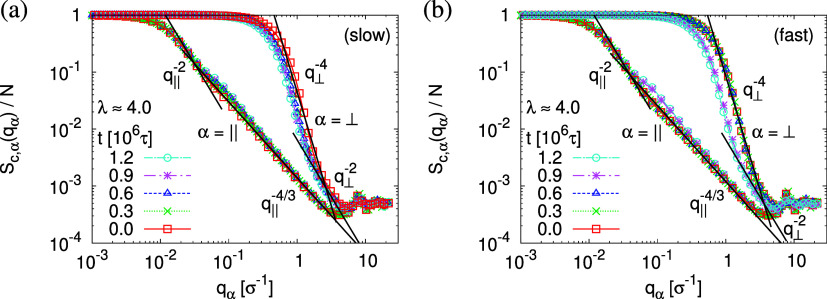
Two components of single chain structure factor *S*_c,α_(*q*) in the directions parallel (α = ∥) and perpendicular (α = ⊥) to the expanding directions for films upon slow (a) and fast (b) expansion. Data for several selected relaxation times *t* are shown, as indicated. Expected scaling laws are shown by straight lines for comparison, cf. text.

This comes along with the restricted conformational relaxation of the individual chains, as characterized by their linear dimensions, and structure factor. The time-dependent two components of ⟨*R*_g,α_^2^(*t*)⟩ parallel (α = ∥) and perpendicular (α = ⊥) to the film interfaces, and the bond orientational order parameter *Q*_λ_(*t*) are shown in Figure 17. ⟨*R*_g,α_^2^(*t*)⟩ only decreases marginally during initial relaxation and then remains almost unchanged, while ⟨*R*_g,⊥_^2^(*t*)⟩ increases slightly with time *t* after a very short time initial decrease for the fast expanded film. Both approach a similar plateau value for longer times. Note that this is an almost marginal relaxation, indicating that the chain retraction inside the tube as predicted by the Doi–Edwards and GLaMM tube models^58,59^ here is strongly retarded. Similarly, the bond orientational order parameter *Q*(*t*) displays a significant relaxation delay toward the isotropic phase. The two components of the single chain structure factor, *S*_c,∥_(*q*_∥_) and *S*_c,⊥_(*q*_⊥_), remain almost unchanged with time *t*, cf. Figure 18. The instantaneously observed crossover from a two-dimensional self-avoiding walk-like structure (*S*_c,∥_(*q*_∥_) ∼ *q*_∥_^–4/3^) to ideal random walk-like structure (*S*_c,∥_(*q*_∥_) ∼ *q*_∥_^–2^) remains and occurs around *q*_∥_ values, where the collective structure factor *S*_∥_(*q*_∥_) shows the plateau/weak maximum. The perpendicular component displays an even slightly more pronounced Porod power law (*S*_⊥_(*q*_⊥_) ∼ *q*_⊥_^–4^), indicating the sharp surface.

### Expanded Films Subject to Cooling

Despite the strongly retarded relaxation, experimentally, systems of course are instable and the nanoporous structure eventually would disappear. A way to stabilize such structures is to quench them down deep into the glassy state. We perform such a quench for the four samples in the thin-film regime marked in Figure 2. We apply a stepwise cooling rate^60^ as in our previous studies^25,31,32,42,55^ Γ = Δ*T*/Δ*t* = 8.3 × 10^–7^ϵ/(*k*_B_τ) with Δ*T* = 0.025ϵ/*k*_B_ and Δ*t* = 30,000τ. In equilibrium, that would allow subchains of up to *N*_s_ ≈ 102σ ≈ 3.6*N*_e_ to relax easily at temperatures close to *T* = 1.0ϵ/*k*_B_. Under these conditions the glass transition temperature is close to *T*_g_^(0)^ = 0.67ϵ/*k*_B_ for bulk melts. We perform MD simulations in the *NVT* ensemble with Langevin thermostat from *T* = 1.0ϵ/*k*_B_ to 0.2ϵ/*k*_B_ using the package ESPResSo++ .^43,44^ Next to λ ≈ 4.0, we also on the side consider λ ≈ 3.0, the expansion ratio, where pore forming instabilities set in.

**Figure 19:**
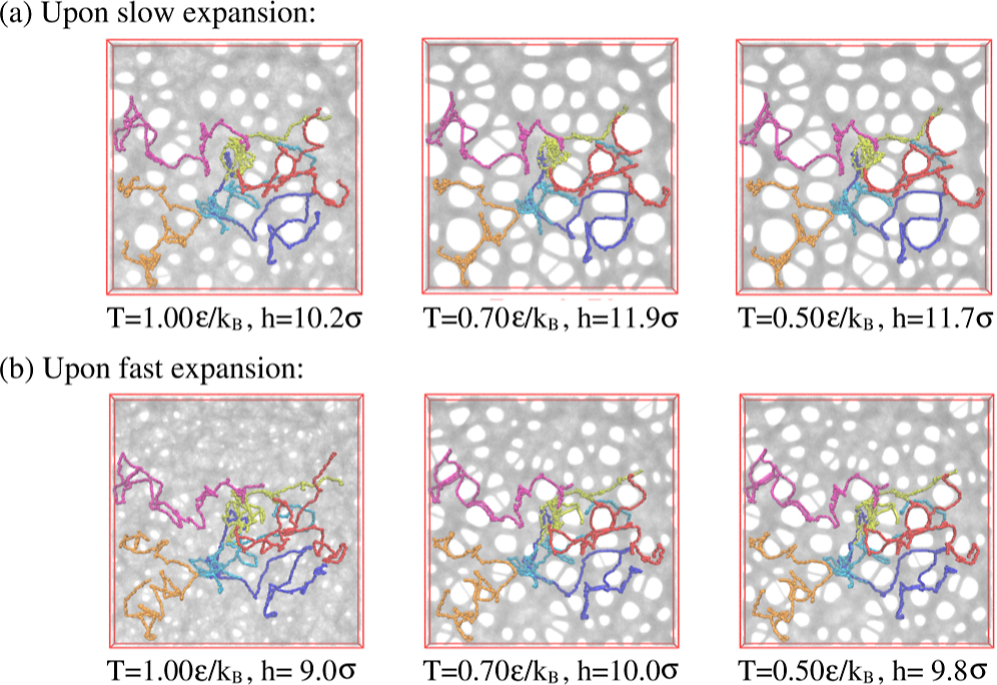
Snapshot configurations at λ ≈ 4.0 subject to cooling for films upon slow (a) and fast (b) expansion at several selected temperatures *T*, as indicated. The very same six chains as shown in Figure 3 are also marked for comparison.

**Figure 20:**
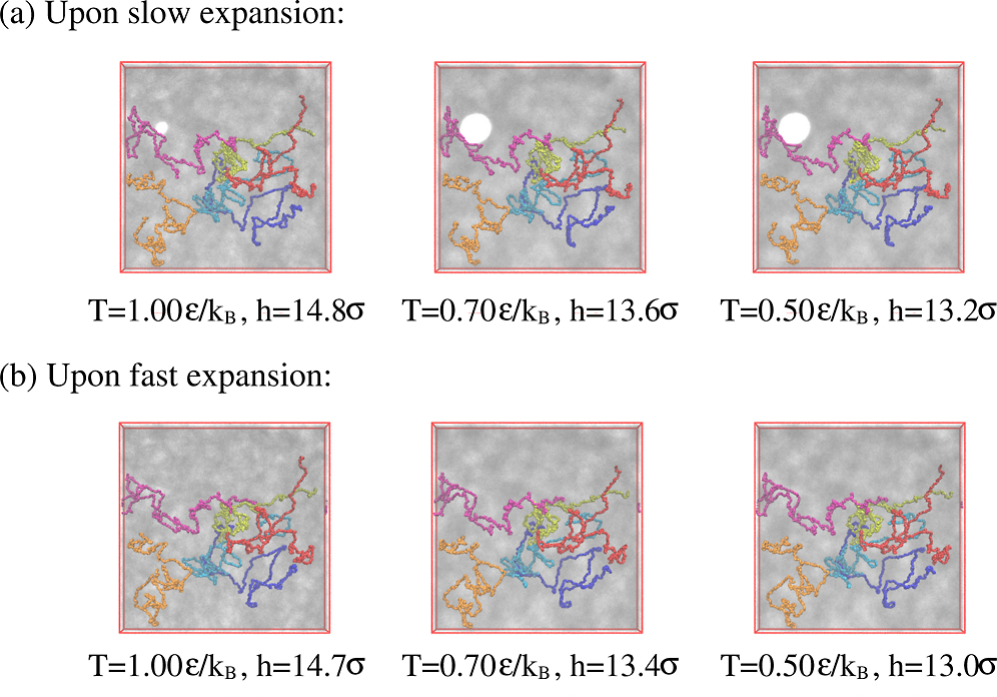
Snapshot configurations at λ ≈ 3.0 subject to cooling for slow (a) and fast (b) expansion at several selected temperatures *T*, as indicated. The very same six chains as shown in Figure 3 are marked for comparison.

Figures 19 and 20 show expanded films at λ ≈ 4.0 and 3.0 at *k*_B_*T*/ϵ = 1.0, 0.7, and 0.5, where cooling started right after the expansion. For λ ≈ 4.0, stable porous structures are already observed at *T* = 0.7ϵ/*k*_B_ due to the competition of very slow large scale chain relaxation and surface minimizing surface tension. Chains extend over several closed pores and the pore pattern is very similar as observed for longer relaxation at higher temperatures. In contrast, for λ ≈ 3.0, the applied load is not sufficient to give the porous structure. Only for slow deformation, we detect just one larger pore, comparable in size to the ones observed for λ ≈ 4.0. To get more insights, we also look at slices of thickness 3.0σ at *T* = 0.5ϵ/*k*_B_. An open network of strands or one single big hole is observed for films at λ ≈ 3.0 (see Figures S12 and S13) while a stable, highly entangled network is formed at λ ≈ 4.0 (see Figures S14 and S15). Thus, we only focus on results at λ ≈ 4.0 hereafter in this section.

**Figure 21:**
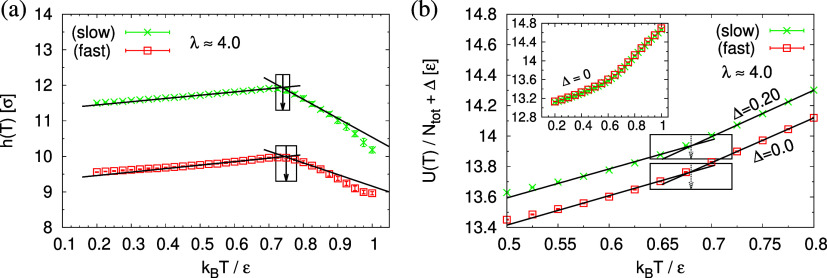
Film thickness *h*(*T*) (a) and total potential energy per monomer *U*(*T*)/*N*_tot_ (b) plotted as a function of temperature *T* as indicated. The straight lines are best fits to the data of the liquid and the glass branch, respectively. Corresponding *T*_g_s are marked by an arrow including the uncertainty. In (b), data for 0.5 ≤ *k*_B_*T*/ϵ ≤ 0.8 are shifted by Δ *f*or better illustration, while the whole range of original data of *U*(*T*) is shown in the inset.

First, the glass transition temperature *T*_g_ is determined from the intersection of linear extrapolation of the film thickness *h*(*T*) estimated from the monomer density profile ρ(*z*) (see Figure S16 of the Supporting Information), *T*_g,*h*,λ_^(slow)^, (*T*_g,*h*,λ_^(fast)^), and the total potential energy *U*(*T*), *T*_g,*U*,λ_^(slow)^, (*T*_g,*U*,λ_^(fast)^), between the liquid branch and the glass branch for slow (fast) expanded films as shown in Figure 21. Though these estimates differ somewhat, as given in Table 2, they indicate that the films become glassy weakly above the bulk glass transition. This is in agreement to the observed enhanced friction of the strained films at higher temperatures. Note that here the surface effect due to the pore formation is not taken into account, and the density is estimated by (*n*_c_*N*)/*V*_film_.

**Table 2:** Estimates of *T*_g_ [ϵ/*k*_B_] via the Change in the Total Potential Energy *U*(*T*) and Film Thickness *h*(*T*) for Expanded Polymer Films at λ ≈ 4.0^a^

	*T*_g,*U*,λ_^(slow)^	*T*_g,*U*,λ_^(fast)^	*T*_g,*h*,λ_^(slow)^	*T*_g,*h*,λ_^(fast)^	*T*_g_^(0)^
	0.68(4)	0.68(4)	0.74(2)	0.75(3)	0.6718(44)

a The bulk value^42^*T*_g_^(0)^ is also given for comparison.

**Figure 22:**
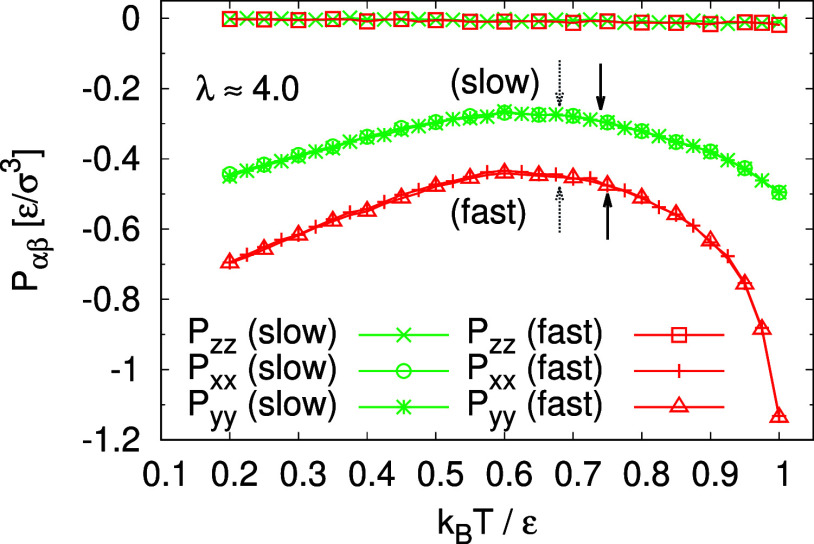
Diagonal terms of pressure tensor *P*_αβ_ versus temperature *T* upon slow and fast expansion, as indicated. *T*_g_ determined from the change of the total potential energy *U*(*T*) and film thickness *h*(*T*) (see Figure 21) are indicated by gray and black arrows, respectively.

At the same time, the pressure, eq S2, along the direction perpendicular to the film remains at *P*_*zz*_ ≈ 0.0ϵ/σ^3^, as expected for freestanding films, see Figure 22. The restoring force of the strained films, given by a negative in-plane pressure, first reduces but does not reach zero. Actually weakly below *T*_g_, it appears that the restoring force (−*P*_*xx*,*yy*_) passes through a minimum. Qualitatively, this is the same for both expansion rates, with a smaller restoring force for the slower expansion case. This, however, does not mean that the films are instable, if the lateral dimensions are free to adjust. *L*_*x*,*y*_ only shrink by about 1% to compensate the remaining in-plane stress and −*P*_*xx*,*yy*_ ≈ 0ϵ/*k*_B_, cf. Figure S17 of the Supporting Information. Thus, in the glassy state, film morphologies are frozen in the state determined by the entanglement structure at higher temperatures.

**Figure 23:**
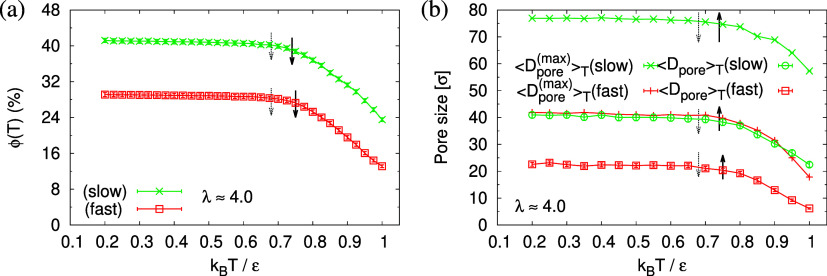
Porosity ϕ(*T*) (a) mean pore size  and mean maximum pore size  (b) plotted as a function of temperature *T* for expanded thin films, as indicated. Estimates of *T*_g_ from *h*(*T*) and *U*(*T*) are indicated by solid black and dotted gray arrows for each sample, respectively, cf. Figure 21.

The slowing down of relaxation, as observed already at *T* = 1.0ϵ/*k*_B_, is even more pronounced at reduced temperatures and the systems seem not to change any more around and below *T* = 0.7ϵ/*k*_B_. In agreement to the monomer density (cf. Figure S16 of the Supporting Information), the temperature-dependent porosity ϕ(*T*) and pore size , Figure 23, stabilize around that temperature. Both first increase with the decrease of *T* for *T* > *T*_g_ and then approach a plateau value, which remains constant at lower temperatures. As expected, the cooling process stabilizes thin porous films and depending on the expansion rate, porosity and pore size of the films can be adjusted. We find ϕ(*T*) ≈ 41%, ,  (slow) and ϕ(*T*) ≈ 29%, ,  (fast) for *T* ≪ *T*_g_. The pore size distribution *P*(*D*_pore_) presented in Figure S18 of the Supporting Information shows similar behavior as observed in Figure 15. For the slowly expanded film, the pore size distribution is much broader, and larger pores are formed.

**Figure 24:**
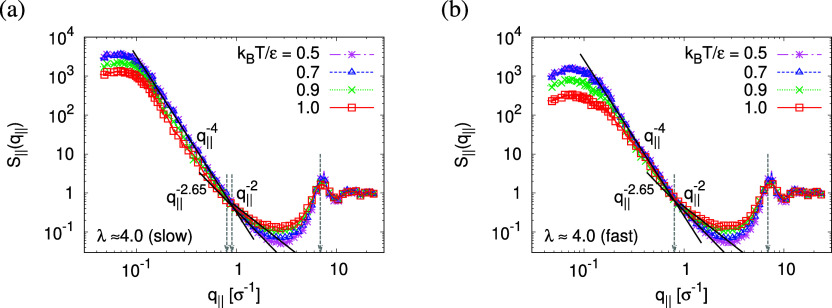
Collective structure factor *S*_∥_(*q*_∥_) in the directions parallel to the interface of expanded thin films at λ ≈ 4.0 upon slow (a) and fast (b) expansion, plotted versus the wave factor *q*_∥_ at several selected temperatures *T*, as indicated. Theoretical predictions are shown by solid straight lines for comparison. The positions of amorphous halo and crossover points are indicated by arrows.

The temperature dependence of the collective structure factor in the expanding direction, *S*_∥_(*q*_∥_), supports the previous findings as shown in Figure 24. For *T* ≤ 0.7ϵ/*k*_B_, no changes are observed anymore for both slow and fast expansion. As for relaxation at *T* = 1.0ϵ/*k*_B_, see Figure 16, the local bead packing is not affected and the peak of the amorphous halo at *q*_∥_ = *q*_∥_^*^ ≈ 6.9σ^–1^ remains essentially unchanged. With decreasing *T*, only a minor sharpening is observed. The curves of *S*_∥_(*q*_∥_) level off in a broad maximum/shoulder below. For small values of *q* below *q* = 0.1σ^–1^ (slow), 0.2σ^–1^ (fast) corresponding to the upper limit of pore diameters, Figure 23b, the shallow maximum now is a bit more pronounced than for *T* = 1.0ϵ/*k*_B_. On intermediate length scales, the sharp pore surfaces are well represented by Porod’s law scaling *S*_∥_(*q*_∥_) ∼ *q*_∥_^–4^. On even shorter length scales, but well above the amorphous halo, a shift to *q*_∥_^–2.65^ at *T* < *T*_g_ instead of *q*_∥_^–2^ at *T* = 1.0ϵ/*k*_B_ is observed. This is expected to be a combined result of local wall structure and microscopic monomer packing. In ref (25), we have demonstrated that this is in qualitative agreement with scattering experiments well below *T*_g_.

## Conclusions

In summary, we have demonstrated that polymer entanglements make it possible to create thin nanoporous polymer films simply by biaxial expansion of thick freestanding films. No additional chemical processing or stabilization beyond quenching them into the glassy state is needed. Even well above *T*_g_, relaxation of the instable films is slowing down dramatically due to enhanced bead friction, which is due to the clustering of entanglement points, as will be shown in a separate publication. The glass transition temperature itself for films at strain of λ ≈ 4.0 remains within our error bars at the bulk value. Slower expansion is found to lead in average to larger pores, while faster expansion results in smaller pores but an overall higher porosity. At the same time, the surface of expanded films is rougher for films upon fast expansion. In general, the pore diameter is determined by the entanglement structure of the polymer melts, leading to pore diameters in average of about 4–10 times the tube diameter *d*_T_ for the present set of parameters. Experimentally, such a clear separation of relaxation conditions as studied here is very difficult to achieve. Furthermore, temperature quenches in simulation are much faster than in experiment. However, our finding that even at *T* = 1.0ϵ/*k*_B_, relaxation is drastically slowed down and that qualitatively low and high *T* data agree suggests the present process to be rather robust also experimentally. Motivated by that, we have shown recently that nanoporous monodisperse PS films can be generated and stabilized experimentally by an analogous procedure.^25,26^ The single chain structures in expanded thin films and details of entanglement effects will be discussed in forthcoming work.

## Data Availability

The data that support the findings of this study are available from the authors upon reasonable request.

## Supporting Information Available

Detailed expansion protocol, definition of pressure tensor, additional results for the film subject to fast expansion, method for estimating porosity and pore size, and internal structures of thin expanded films (PDF)
