# Supplementary material for: Entanglement-Stabilized Nanoporous Polymer Films Made by Mechanical Deformation
Source: Macromolecules. 2024 Mar 14;57(6):2998–3012. doi: 10.1021/acs.macromol.4c00187 (PMC10976899; doi:10.1021/acs.macromol.4c00187)
Supplement: Supplementary file 1 — ma4c00187_si_001.pdf [file ma4c00187_si_001.pdf]

Supporting Information

Entanglement stabilized nanoporous polymer films made by mechanical deformation

Hsiao-Ping Hsu* and Kurt Kremer†

Max-Planck-Institut für Polymerforschung, Ackermannweg 10, 55128, Mainz, Germany

SI. DYNAMIC PROPERTIES OF EQUILIBRATED POLYMER MELTS

The dynamic behavior of polymer chains of chain length N in a melt is usually characterized by the mean square displacement (MSD) of monomers. The theoretical predictions of the dynamic scaling behavior of MSD given by the Rouse model [1], and reptation theory [2, 3] are illustrated in Figure S1.

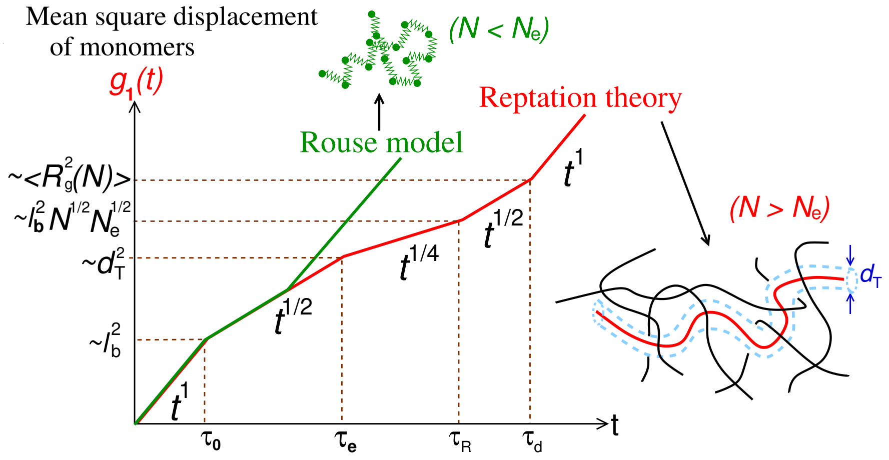

Figure S1. A schematic drawing of MSD (usually called $g_1(t)$) of monomers. The crossover points between two different scaling regimes correspond to the characteristic microscopic relaxation time τ_0, the entanglement time $\tau_e = \tau_0 N_e^2$ (N_e being the entanglement length), the Rouse time $\tau_R = \tau_0 N^2$, and the reptation (disentanglement) time $\tau_d = \tau_0 N^2 (N/N_e)^{1/4}$. Between τ_e and τ_R, monomers are restricted to move only along an imaginary tube, which is geiven by the averaged contour of the very chain. The tube diameter $d_T \propto N_e^{1/2}$ and its contour length $L_T = d_T(N/N_e)$. $R_g(N)$ and l_b denote the radius of gyration of chains and bond length between monomers, respectively.

* hsu@mpip-mainz.mpg.de
† kremer@mpip-mainz.mpg.de

SII. TWO INTRODUCED POTENTIALS IN A NEW VARIANT OF BEAD-SPRING MODEL

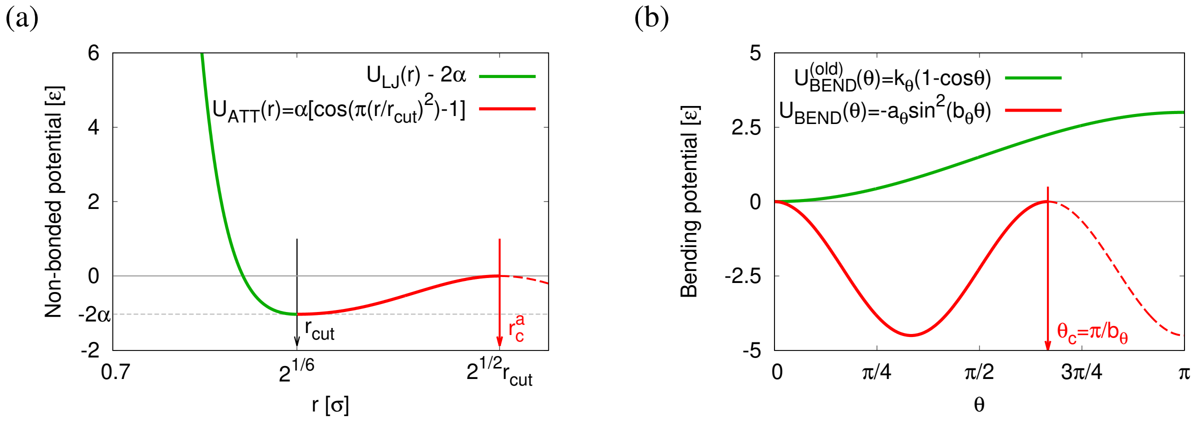

Figure S2. (a) Short-range purely repulsive Lennard-Jones and attractive potentials, $U_{\rm LJ}(r)$ and $U_{\rm ATT}(r)$ with $\alpha = 0.5145\epsilon$, plotted as a function of distance r between non-bonded monomers. $U_{LJ}(r) - 2\alpha = U_{\rm ATT}(r)$ at $r = r_{\rm cut} = 2^{1/6}\sigma$ and $U_{\rm ATT} = 0$ at $r = r_c^a = \sqrt{2}r_{\rm cut}$. (b) Standard and new bond-bending potentials, $U_{\rm BEND}^{\rm (old)}(\theta)$ with $k_\theta = 1.5\epsilon$ and $U_{\rm BEND}(\theta)$ with $a_\theta = 4.5\epsilon$ and $b_\theta = 1.5$, plotted as a function of bond angle θ between two successive bonds. The cut-off values are pointed by arrows. Details see Refs [4, 5].

SIII. FREE-STANDING FILM SUBJECT TO BIAXIAL EXPANSION

The expansion protocol is as follows: Starting from a fully equilibrated free-standing films, the film is first instantaneously stretched by a factor of 1.02 along the x, and then along the $y-$ direction with periodic boundary condition along the two lateral dimensions. The strain rate along each direction is defined by

$$\dot{\varepsilon} = \frac{1}{L_{x,y}}\frac{dL_{x,y}}{dt} = \frac{d\ln L_{x,y}}{dt} = \frac{d\ln(L_{x,y}(t)/L_{x,y}(0))}{dt} = \frac{d\varepsilon}{dt}\,, \qquad \text{i.e., } L_{x,y}(t)/L_{x,y}(0) = \exp(\varepsilon t)\,. \tag{S1}$$

After each instantaneous stretch, the film is relaxed for $dt = 0.02/\dot{\varepsilon} = (0.02\tau_R/C)$ with the strain rate $\dot{\varepsilon} = C/\tau_R$ (τ_R being the Rouse time) we set. Two strain rates $C = 77$ (slow) and 32000 (fast) are chosen such that subchains of chain length $8N_e$ and $0.4N_e$ ($N_e \approx 28$ being the entanglement length), respectively, are relaxed after each instantaneous stretch. This deformation step is so small that it does not induce any instabilities in the simulations and mimics a quasi continuous deformation. Moreover, to stabilize the free surfaces, we keep the lateral dimensions of expanded film after the strain is increased by a factor of $(1.02)^3 \times (1.02)^3$, and let the film relax and adjust its film thickness until the pressure in the direction perpendicular to the interfaces, $P_{zz} \approx 0.0\epsilon/\sigma^3$. We repeat this same procedure up to a strain of $4 \times 4 \approx (1.02)^m \times (1.02)^m$ with $m = 69$, where the resulting expanded film is in the thin film regime, i.e., the film thickness $h < R_g^{(0)}$ (e.g. Figure S4a). Here $R_g^{(0)} \approx 30.15\sigma$ is the root-mean-square radius of gyration of chains in a fully equilibrated bulk melt containing $n_c = 1000$ chains of $N = 2000$ monomers [6]. Considering the total relaxation time $t_{\rm stretch} = 133200\tau$ (slow), 2169τ (fast) during the expansion process, which includes the intermediate short pressure relaxations times, the effective average relaxation time for each stretching step is about $dt_{eff} = t_{\rm stretch}/(2m)$, i.e. $\dot{\varepsilon} = C/\tau_R = 0.02/dt_{eff}$. We obtain the average effective strain rate $C \approx 24$ (slow) and $C \approx 14710$ (fast), i.e., on average, subchains of chain lengths $14.6N_e$ (slow) and $0.6N_e$ (fast) are relaxed. According to

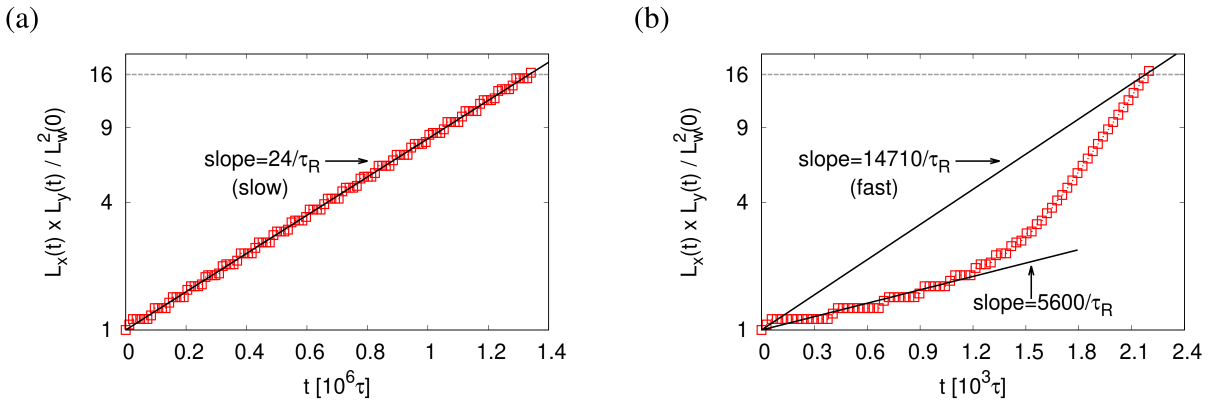

Figure S3. Change in lateral dimensions $(L_x(t) \times L_y(t))/L_w^2$ plotted versus the relaxation time t on a semi-log scale for films subject to slow (a) and fast (b) expansion. The effective strain rate C/τ_R is determined by the slope, see Eq. (S1).

Eq. (S1), the strain rate should also be determined by the fractional change in lateral dimensions, $(L_x(t) \times L_y(t))/L_w^2$ with $L_x(t=0) = L_y(t=0) = L_w \approx 134\sigma$, with respect to the relaxation time t as shown in Figure S3. Indeed, the slope is given by C/τ_R for films subject to slow expansion. For fast expansion, the initial effective strain rate is much slower ($C \approx 2800$ for $\lambda < 1.5$) than the average effective strain rate ($C \approx 14710$), i.e., the required relaxation time for $P_{zz}(t)$ (the retraction force) recovering to zero is longer than the average relaxation time, see Figure S4b.

Considering the pairwise potential U_{ij}, the three-body bond bending potential U_{ijk}, and the total effective volume of film, $V_{\rm film} = hL_xL_y$, here the components of pressure tensor $P_{\alpha\beta} = -\sigma_{\alpha\beta}$ ($\sigma_{\alpha\beta}$ being the components of stress tensor defined via the virial theorem) is given by

$$\begin{aligned} P_{\alpha\beta} = & \frac{1}{V_{\rm film}} \left(\sum_{i=1}^{n_cN} m_i v_i^{(\alpha)} v_i^{(\beta)} + \frac{1}{2} \sum_{i,j=1}^{n_cN} f_{ij}^{(\alpha)} r_{ij}^{(\beta)} \right) \\ & - \frac{1}{6V_{\rm film}} \sum_{i,j,k=1}^{n_cN} \left(\frac{r_{ij}^{(\alpha)} r_{ij}^{(\beta)}}{r_{ij}} \frac{\partial U_{ijk}}{\partial r_{ij}} + \frac{r_{jk}^{(\alpha)} r_{jk}^{(\beta)}}{r_{jk}} \frac{\partial U_{ijk}}{\partial r_{jk}} + \frac{r_{ki}^{(\alpha)} r_{ki}^{(\beta)}}{r_{ki}} \frac{\partial U_{ijk}}{\partial r_{ki}} \right) \end{aligned} \quad \text{(S2)}$$

where m_i and v_i^α are the mass and the αth component of the velocity vector of the ith monomer, respectively for $i = 1, 2, \ldots, n_cN$, $r_{\rm ij} =\mid \vec{r}_j - \vec{r}_i \mid$ is the distance between the ith monomer and the jth monomer, and $f_{ij}^{(\alpha)} \equiv -\frac{r_{ij}^{(\alpha)}}{r_{ij}} \frac{\partial U_{ij}}{\partial r_{ij}}$ is the αth component of the force vector acting on the ith monomer by the jth monomer.

Linear dimensions of a thick free-standing film subject to expansion at a fast effective strain rate $\dot{\varepsilon}\tau_R = C = 14710$, $L_{x,y}(t)$ and $h(t)$, and the resulting three diagonal terms of pressures tensor $P_{\alpha\alpha}(t)$ with $\alpha = x$, y, and z during the expansion process are shown in Figure S4. Results of the rescaled internal mean-square distances right after deformation ($C = 24$) at several selected strain of λ, $C_R \langle R_\alpha^2(n) \rangle_\lambda / \langle R_\alpha^2(n) \rangle_{\lambda=1}$ in Figure S5, normalized by the affine deformation scaling parameter C_R, as indicated. Here n is the chemical distance between two bonds along the path of the same chain.

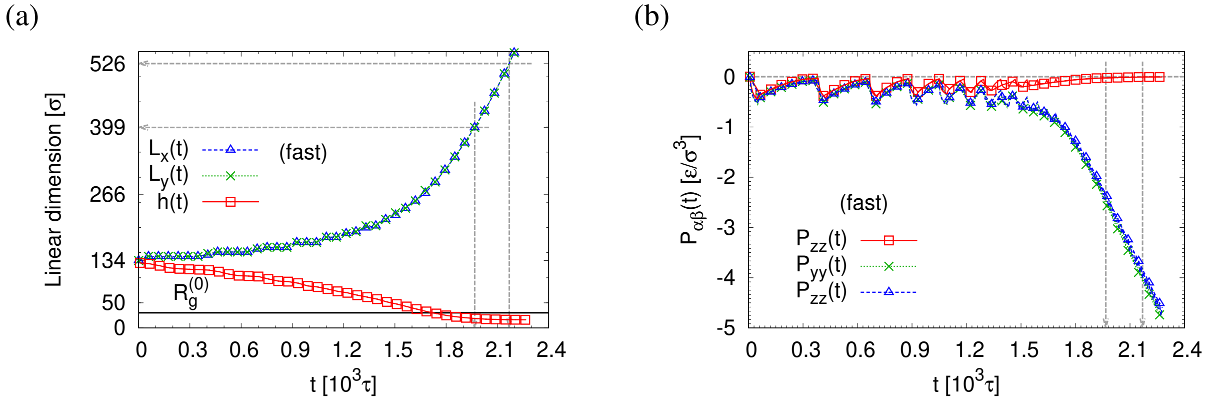

Figure S4. Time series of two lateral dimensions $L_x(t)$, $L_y(t)$, and thickness $h(t)$ of film (a), and three diagonal terms of pressure tensor $P_{\alpha\beta}(t)$ (b) for films subject to fast expansion ($C = 14710$) at $T = 1.0\epsilon/k_B$. The configurations of expanded films of two selected lateral dimensions $L_x(t) = L_y(t) \approx 399\sigma$ and 526σ, i.e. the strain of $\lambda = L_{x,y}/L_w \approx 3.0$ and 4.0, in the thin film regime ($h < R_g^{(0)}$) at $P_{zz}(t) \approx 0.0\epsilon/\sigma^3$ are indicated by arrows for later study.

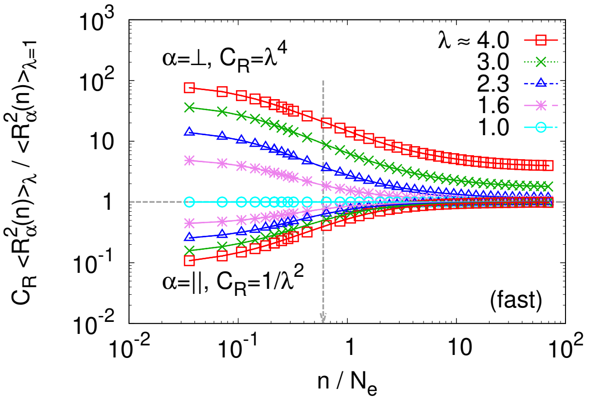

Figure S5. Two components of rescaled mean square internal distance, $C_R\langle R_\alpha^2(n)\rangle_\lambda/\langle R_\alpha^2(n)\rangle_{\lambda=1}$, plotted versus the rescaled chemical distance n/N_e for films subject to fast expansion at several selected strain of λ, as indicated. Subchains of chain length $n = 0.6N_e$ estimated for $C = 14710$ is pointed out by an arrow, cf. main text.

Detailed internal structures of expanded films cut into slices of thickness 3.0σ at $\lambda \approx 3.0$, and 4.0 upon slow and fast expansion are shown in Figures S6-S9.

Upon slow expansion:

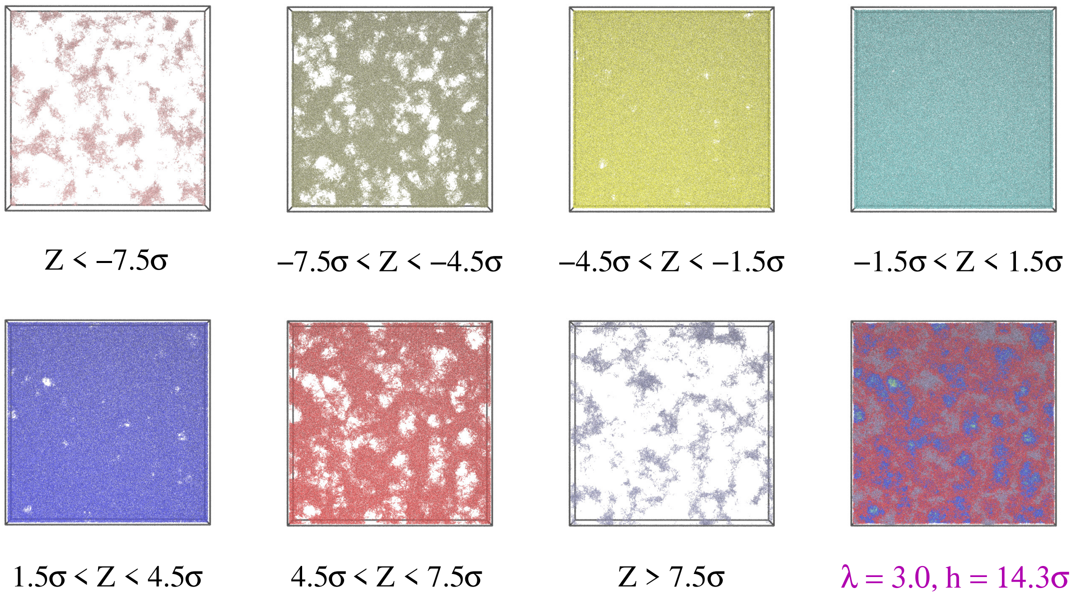

Figure S6. Snapshot configuration of expanded film of $h \approx 14.3\sigma$ at $\lambda \approx 3.0$, $T = 1.0\epsilon/k_B$, cut into seven slices.

Upon fast expansion:

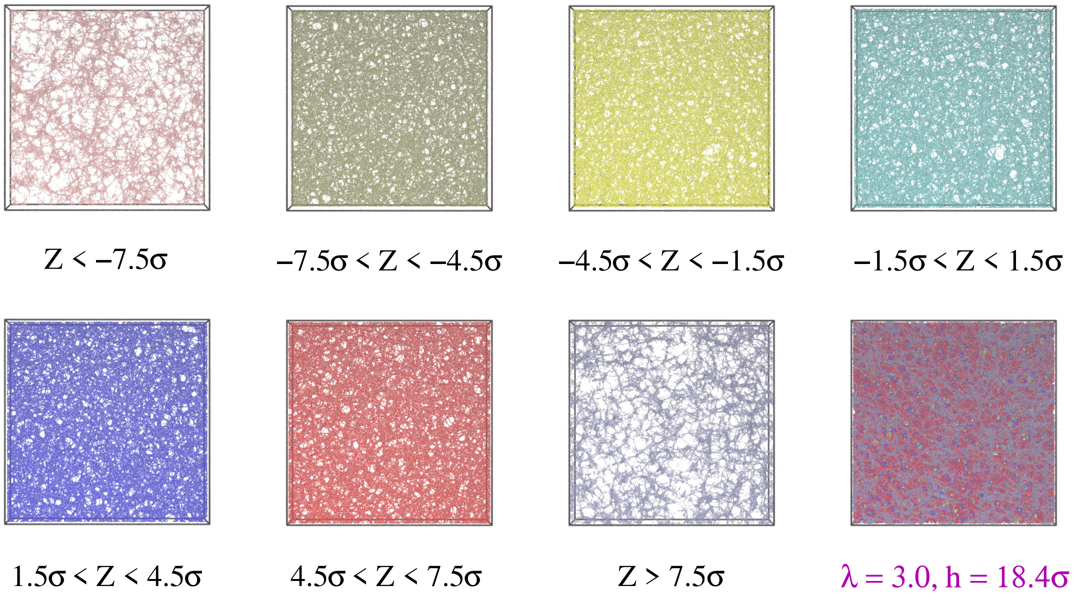

Figure S7. Snapshot configuration of expanded film of $h \approx 18.4\sigma$ at $\lambda \approx 3.0$, $T = 1.0\epsilon/k_B$, cut into seven slices.

Upon slow expansion:

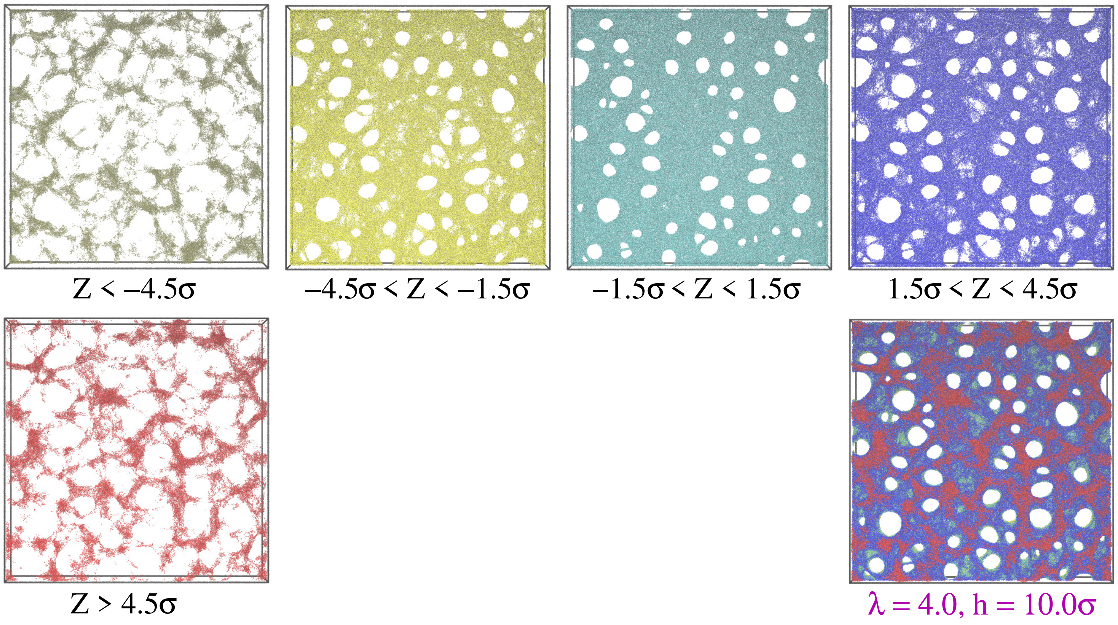

Figure S8. Snapshot configuration of expanded film of $h \approx 10.0\sigma$ at $\lambda \approx 4.0$, $T = 1.0\epsilon/k_B$, cut into five slices.

Upon fast expansion:

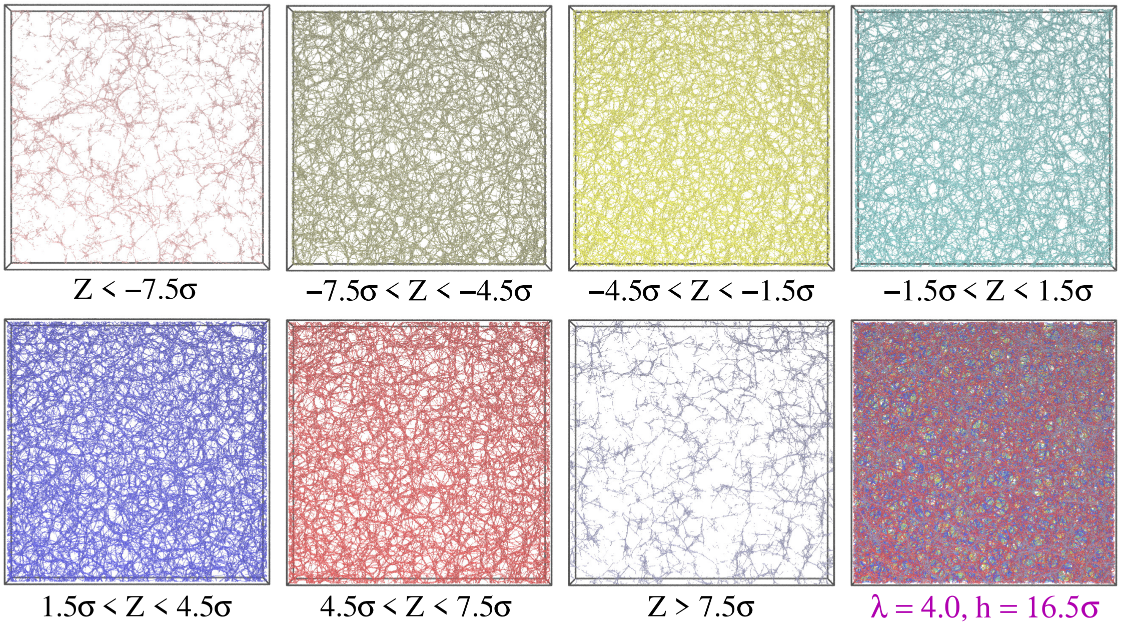

Figure S9. Snapshot configuration of expanded film of $h \approx 16.5\sigma$ at $\lambda \approx 4.0$, $T = 1.0\epsilon/k_B$, cut into seven slices.

Snapshot configurations illustrating the formation of a typical pore is shown in Figure S10.

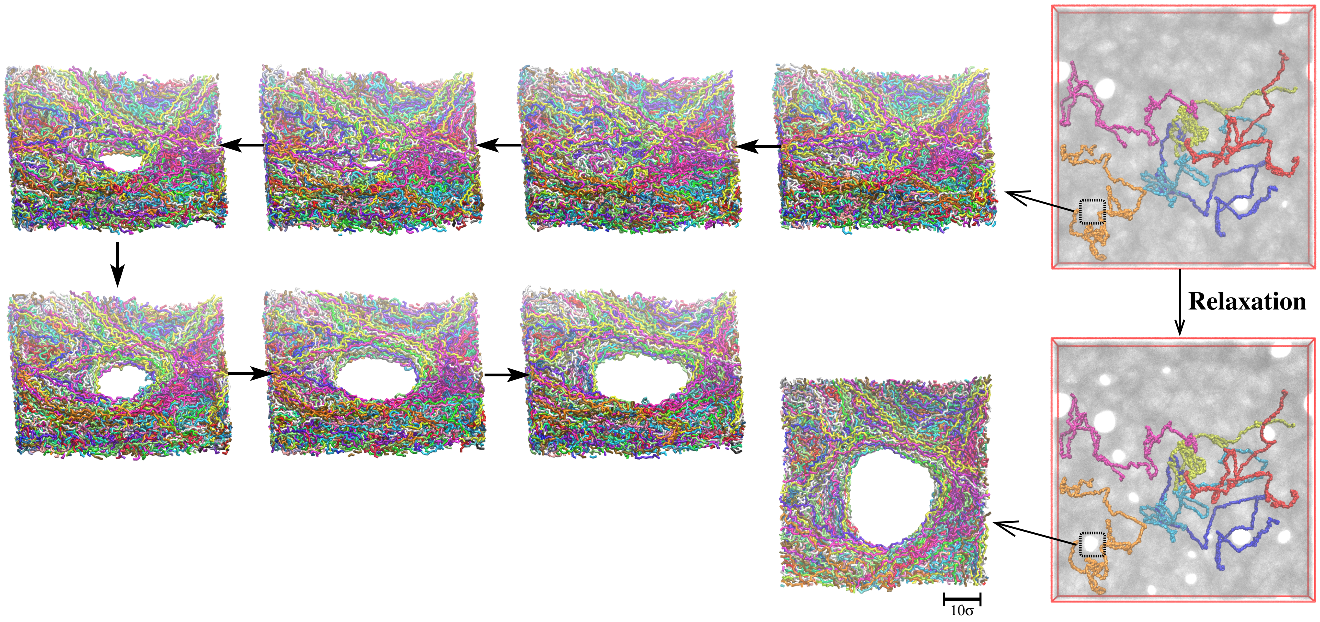

Figure S10. Snapshot configurations of an expanded film at fixed strain of $\lambda \approx 3.5$ before and right after relaxation where the film thickness h freely adjusted from 11.0σ to 11.3σ. The formation process of a marked pore having a spherical-like cross section is illustrated by the changes of local structures from a different perspective.

SIV. ESTIMATES OF POROSITY AND PORE SIZE DISTRIBUTION

The porosity in expanded film is defined by

$$\phi = \frac{V_{\text{void}}}{V_{\text{film}}} \times 100\% \tag{S3}$$

with

$$V_{\text{film}} = hL_xLy \qquad \text{and} \qquad V_{\text{void}} = \int_{z_G^{(\text{lower})}}^{z_G^{(\text{upper})}} dz \int_0^{L_y} dy \int_0^{L_x} dx \; \Theta(d = d_{ik} - 1\sigma) \tag{S4}$$

where $d_{ik} = \mid \vec{r}_k - \vec{r}_i \mid$ is the distance between monomer i and the test particle for $i = 1, 2, \ldots, N_{\text{tot}} (= n_c N)$ and $\Theta(d)$ is the Heaviside step function. Here the effective film thickness $h = z_G^{(\text{upper})} - z_G^{(\text{lower})}$ of expanded film is determined from the monomer density distribution $\rho(z)$ in the direction perpendicular to the interfaces located at $z_G^{(\text{upper})}$ and $z_G^{(\text{lower})}$ as mentioned in the second section (main text), see Figures 1 and 6. Practically, we estimate ϕ of our systems by simply performing a Monte Carlo (MC) integration of V_{void}. The radius R of any pore, assuming a spherical shape of diameter $D_{\text{pore}} = 2R$ in the void space is determined by first randomly selecting a point $\vec{r}_p$ in the void space, and then find the largest radius R of hard sphere located at $\vec{r}_c$ containing $\vec{r}_p$ in the void space satisfying the following conditions: [7, 8]

$$R(\vec{r}_c) = \text{Min.}(d_{0c}, d_{1c}, \ldots, d_{N_{\text{tot}}c}) - \frac{1}{2}\sigma \quad \text{and} \quad \mid \vec{r}_c - \vec{r}_p \mid \leq R(\vec{r}_c)\,, \tag{S5}$$

where $d_{ic} = \mid \vec{r}_c - \vec{r}_i \mid$ is the distance between monomer i and the center of sphere, $\vec{r}_c$. The distribution $P(D_{\text{pore}}) = -\frac{dH(D_{\text{pore}})}{dD_{\text{pore}}}$ is thus given by the negative derivative of the cumulative histogram $H(D_{\text{pore}})$ that counts the probability of finding a point in the void space with a pore size equal and smaller than D_{pore}. We combine the grid-based method [9] with the grid spacing $l_c = 0.8\sigma$ and the Hoshen-Kopelman method [10] in MC simulations to look for unoccupied grid sites in the void space and find the maximal pore radius R according to the criterion given in Eq. (S5). The maximum pore size $D_{\text{pore}}^{(\text{max})} = \text{max.}\{\text{all } D_{\text{pore}} \text{ values in one Monte Carlo block}\}$. Typical snapshot configurations of a free-standing porous film and the corresponding unoccupied grid sites are shown in Figure S11. Choosing $l_c = 0.8\sigma$, pores are well represented by clusters of free grid sites as shown in Figure S11b. However, cutting expanded films into slices, we see that not all pores are permeable, see Figures S8, S9, S14, and S15. Pores can either form in the surface or in the interior of films.

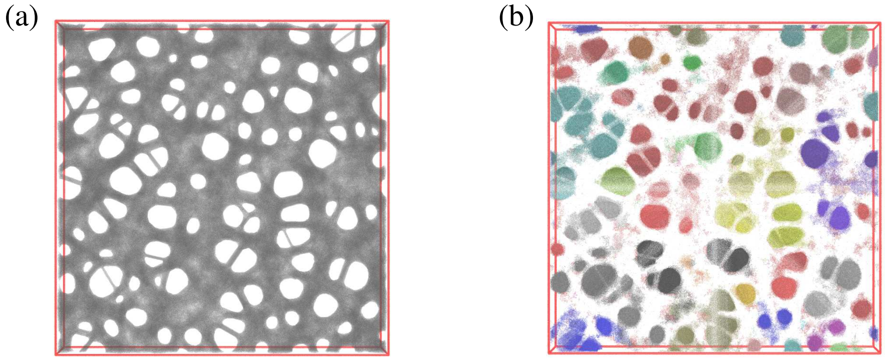

Figure S11. Typical snapshot of a free-standing porous film of lateral dimensions $L_x = L_y \approx 526\sigma$, and film thickness $h \approx 9.7\sigma$ (a), and the corresponding clusters (pores) of unoccupied grid sites (b). Detailed structure in the interior of film, see Figure S15.

SV. EXPANDED FILMS SUBJECT TO COOLING

Expanded films at $\lambda \approx 3.0$ and 4.0 are cooled at a fixed cooling rate $\Gamma = 8.3 \times 10^{-7}\epsilon/(k_B\tau)$. The detailed internal structure of expanded films at $T = 0.5\epsilon/k_B$ below T_g are shown in Figures S12-S15.

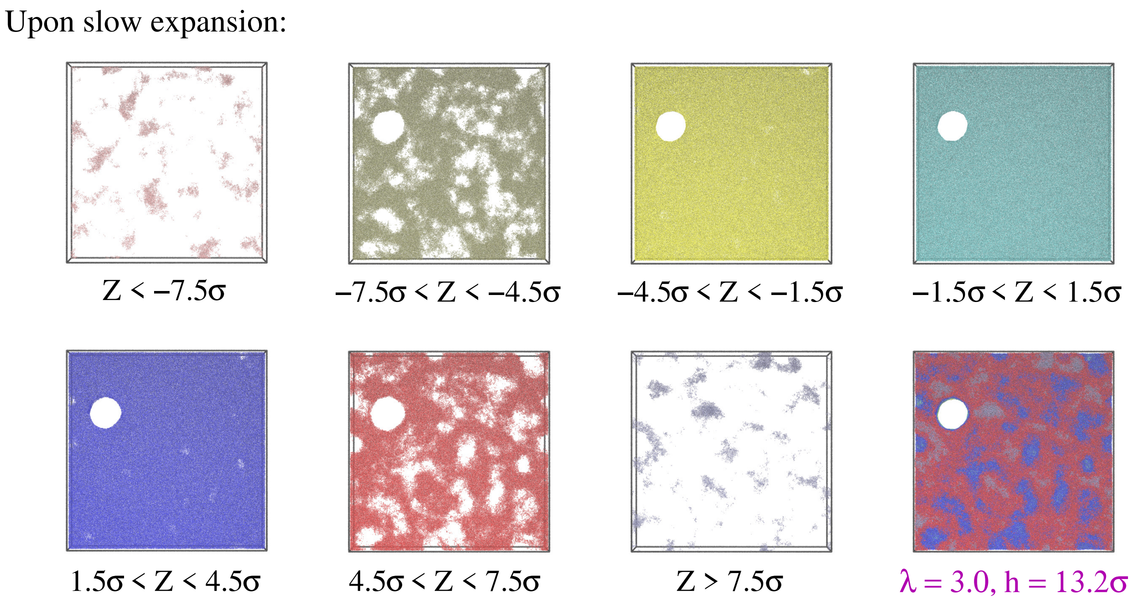

Figure S12. Snapshot of the expanded film of $h \approx 13.2\sigma$ at $\lambda \approx 3.0$, $T = 0.5\epsilon/k_B$, cut into seven slices.

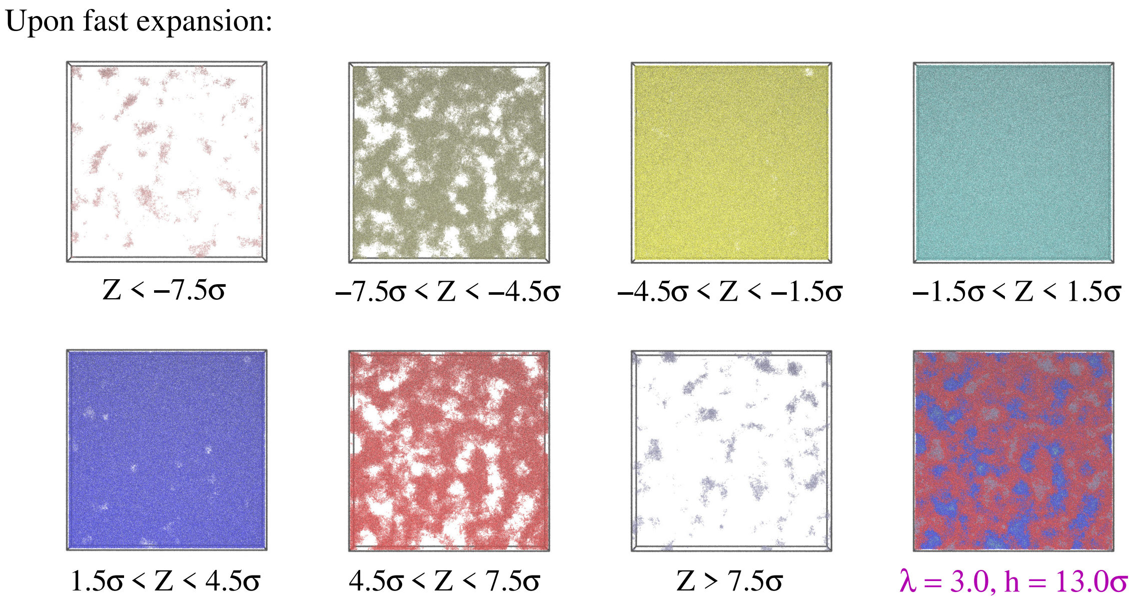

Figure S13. Snapshot of the expanded film of $h \approx 13.0\sigma$ at $\lambda \approx 3.0$, $T = 0.5\epsilon/k_B$, cut into seven slices.

Upon slow expansion:

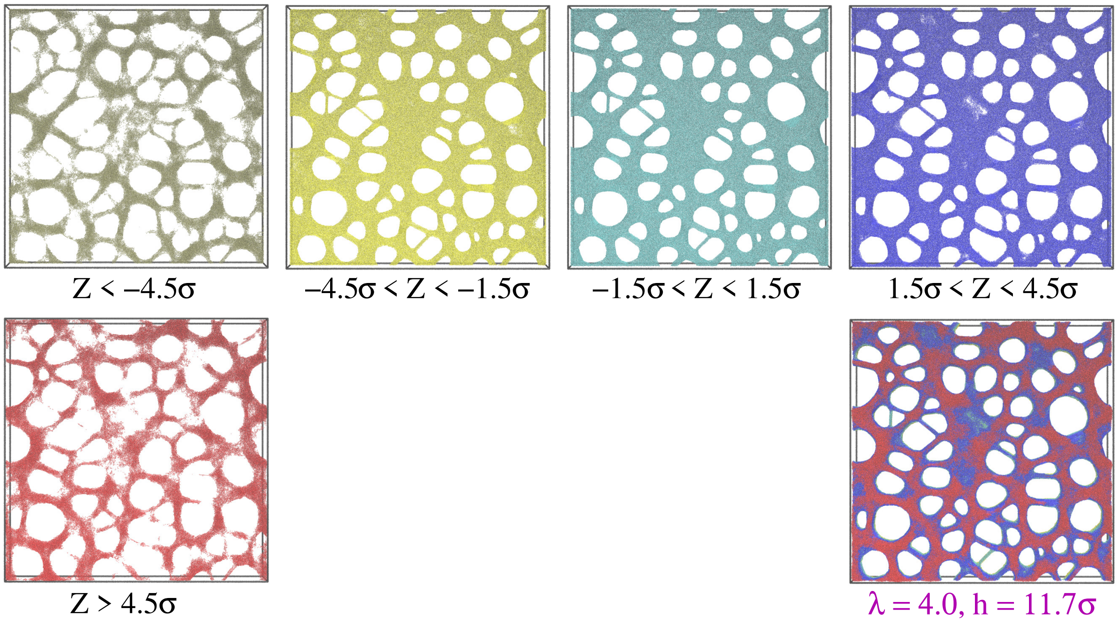

Figure S14. Snapshot of the expanded film of $h \approx 11.7\sigma$ at $\lambda \approx 4.0$, $T = 0.5\epsilon/k_B$, cut into five slices.

Upon fast expansion:

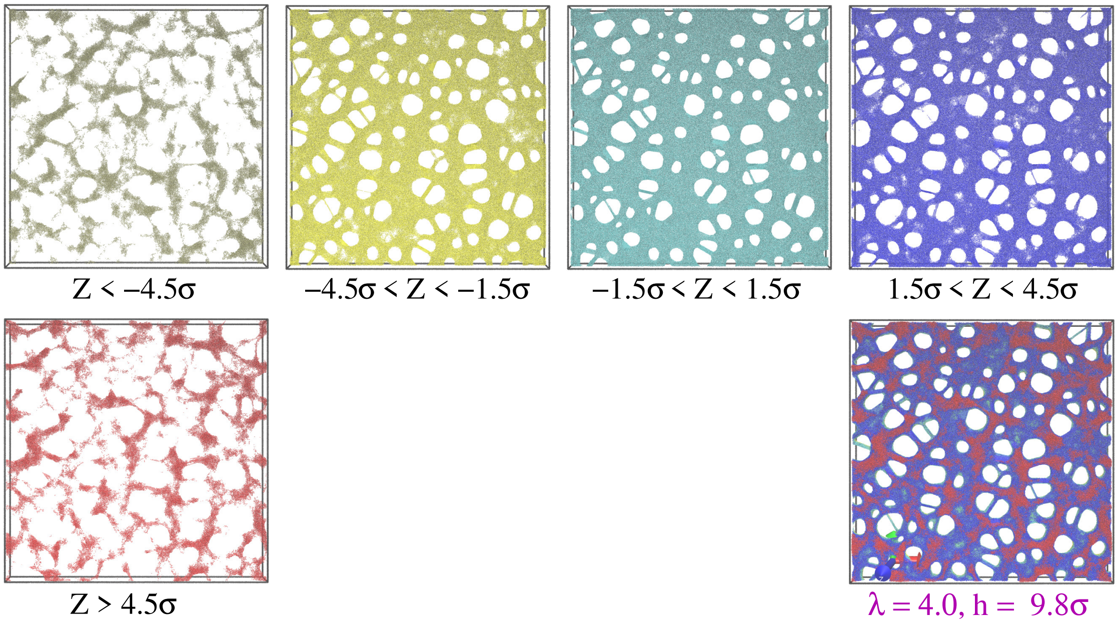

Figure S15. Snapshot of the expanded film of $h \approx 9.8\sigma$ at $\lambda \approx 4.0$, $T = 0.5\epsilon/k_B$, cut into five slices.

The monomer density $\rho(z)$ at several selected temperatures T and the pore size distribution $P(D_{\text{pore}})$ at $T = 1.0\epsilon/k_B$ and $0.5\epsilon/k_B$ are presented in Figures S16 and S18, respectively.

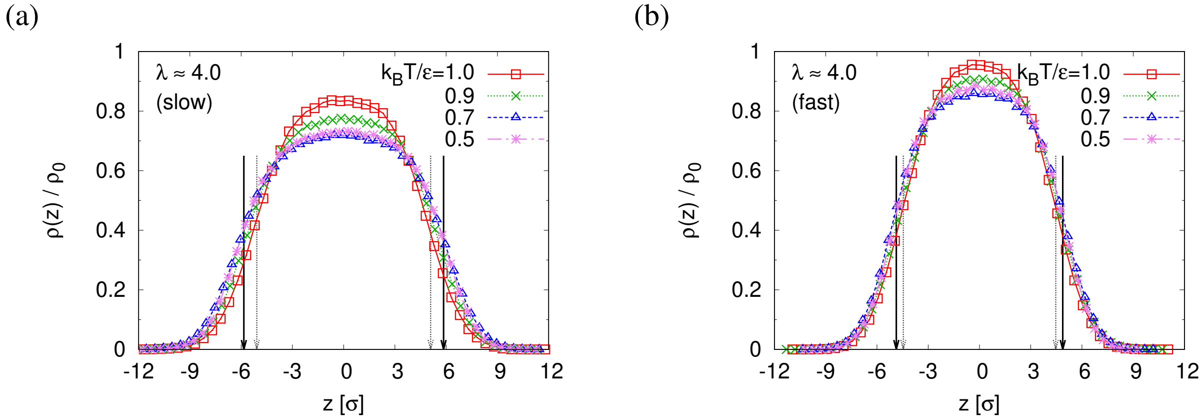

Figure S16. Rescaled monomer density profiles, $\rho(z)/\rho_0$, plotted as a function of z, perpendicular to the interfaces at $\lambda \approx 4.0$ upon slow (a), and fast (b) expansion at several selected temperatures T. The centers of thin porous films in the z-direction are matched at $z = 0\sigma$. The interfaces located at $Z_G^{(\text{lower})}$ and $Z_G^{(\text{upper})}$ are indicated by dashed and solid arrows at $k_BT = 1.0$, and 0.5, respectively.

To investigate the variation in lateral dimensions as the restoring force approaches zero, i.e., $P_{xx,yy} \approx 0.0\epsilon/\sigma^3$, we relax expanded films at $T = 0.4\epsilon/k_B < T_g$ starting from the final configurations of expanded films subject to cooling by performing molecular dynamics simulations in the NPT ensemble. The percentage change in the lateral dimensions $\lambda_p(t)/\lambda \times 100$ % versus the relaxation time t is shown in Figure S17, where $\lambda_p = L_{x,y}(t)/L_w$ with $L_w \approx 526\sigma$.

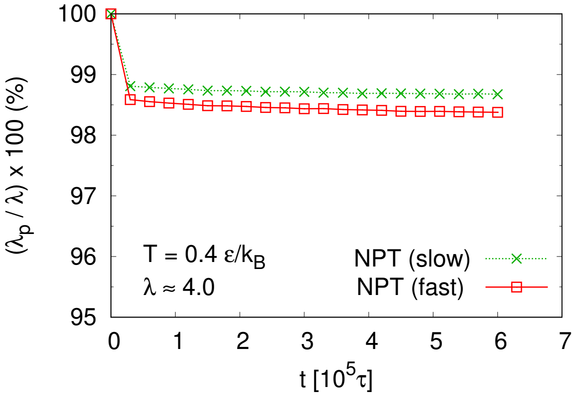

Figure S17. Time series of percentage change in the lateral dimensions, $\lambda_p/\lambda \times 100$ % for expanded film at $\lambda \approx 4.0$ at $T = 0.4\epsilon/k_B$ upon relaxation.

The pore size distributions $P(D_{\rm pore})$ at the temperatures $T = 1.0\epsilon/k_B > T_g$, and $T = 0.5\epsilon/k_B < T_g$ are shown in Fig. S18.

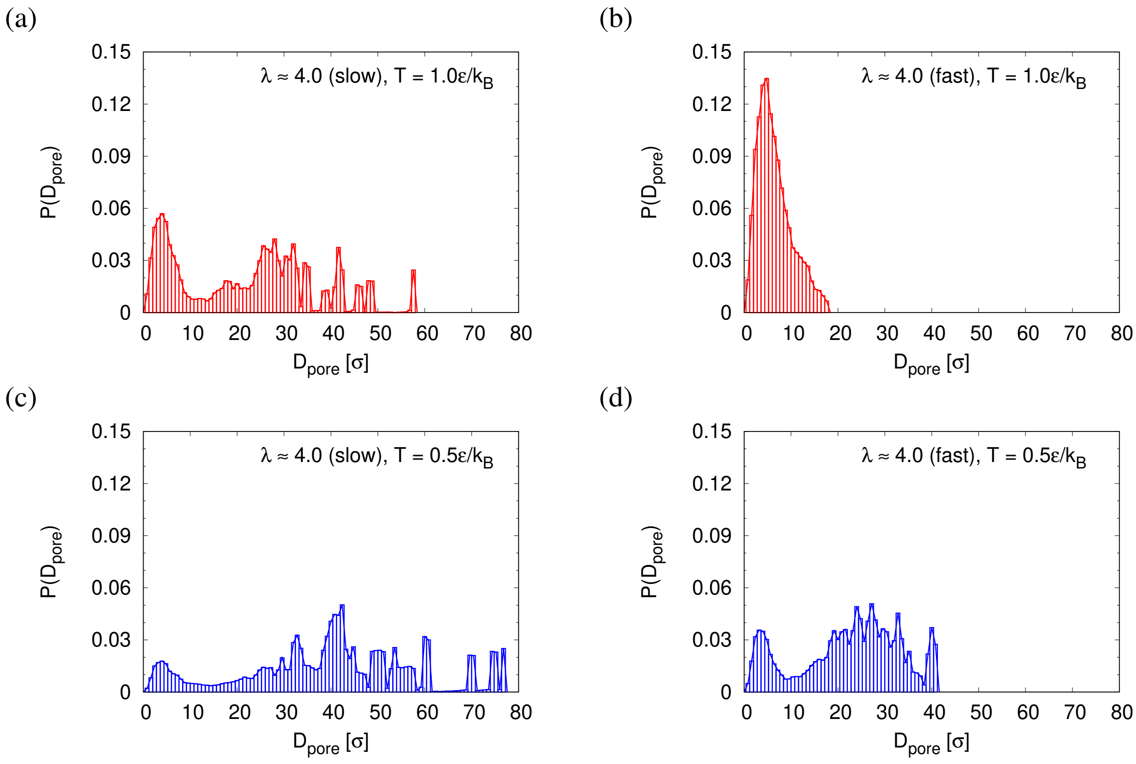

Figure S18. Histogram plot of pores size distribution $P(D_{\rm pore})$ at $T = 1.0\epsilon/k_B$ (a)(b) and $T = 0.5\epsilon/k_B$ (c)(d) right after relaxing for 30000τ at each T for thin porous films at $\lambda \approx 4.0$ upon slow (a)(c), and fast (b)(d) expansion subject to cooling.

[S1] P. E. Rouse, J. Chem. Phys. **21**, 1272 (1953).

[S2] P. G. de Gennes, *Scaling Concepts in polymer physics* (Cornell University Press: Itharca, New York, 1979).

[S3] M. Doi and S. Edwards, *The theory of polymer dynamics* (Oxford University Press, New York, 1986).

[S4] H.-P. Hsu and K. Kremer, J. Chem. Phys. **150**, 091101 (2019).

[S5] H.-P. Hsu, T. Stuehn, K. C. Daoulas, and K. Kremer, in *NIC Symposium 2022 -Proceedings*, edited by M. Müller, C. Peter, and A. Trautmann (Forschungszentrum Jülich GmbH Zentralbibliothek, Verlag, NIC Series, Jülich, Germany, 2022) pp. 135–144.

[S6] H.-P. Hsu and K. Kremer, J. Chem. Phys. **144**, 154907 (2016).

[S7] S. Bhattacharya and K. E. Gubbins, Langmuir **22**, 7726 (2006).

[S8] V. Sorichetti, V. Hugouvieux, and W. Kob, Macromolecules **53**, 2568 (2020).

[S9] D. Hofmann, M. Heuchel, Y. Yampolskii, V. Khotimskii, and V. Shantarovich, Macromolecules **35**, 2129 (2002).

[S10] J. Hoshen and R. Kopelman, Phys. Rev. B **14**, 3438 (1976).
